# Low-carbohydrate diets reduce cardiovascular risk factor levels in patients with metabolic dysfunction-associated steatotic liver disease: a systematic review and meta-analysis of randomized controlled trials

**DOI:** 10.3389/fnut.2025.1626352

**Published:** 2025-08-26

**Authors:** Shanshan Pi, Shuwen Zhang, Junjie Zhang, Yi Guo, Yue Li, Jinyan Deng, Hongbo Du

**Affiliations:** ^1^Department of Gastroenterology, Dongzhimen Hospital, Beijing University of Chinese Medicine, Beijing, China; ^2^Beijing University of Chinese Medicine, Beijing, China; ^3^Department of Cardiology, Beijing Hospital of Traditional Chinese Medicine, Capital Medical University, Beijing, China; ^4^Liver Diseases Academy of Traditional Chinese Medicine, Beijing University of Chinese Medicine, Beijing, China

**Keywords:** low-carbohydrate diet, nonalcoholic fatty liver disease, metabolic dysfunction-associated steatotic liver disease, metabolic dysfunction-associated fatty liver disease, cardiovascular risk factor

## Abstract

**Background:**

Low-carbohydrate diets (LCDs) are increasingly advocated for the treatment of metabolic dysfunction-associated steatotic liver disease (MASLD); however, their cardiovascular safety profile remains controversial. This analysis aims to evaluate the effects of LCDs on cardiovascular risk factors in MASLD patients.

**Methods:**

PubMed, Cochrane Library, Web of Science, and Scopus were searched from inception to March 19, 2025. Two reviewers independently conducted data extraction. Meta-analyses were performed using fixed-effects or random-effects models, as determined by the heterogeneity of the included studies. Outcomes included blood pressure, glycemic markers, lipid profiles, and anthropometric indicators. Subgroup analyses explored carbohydrate thresholds (<26% vs. ≥26%) and intervention durations (<24 weeks vs. ≥24 weeks).

**Results:**

Sixteen RCTs comprising 1,056 participants were included. LCDs significantly reduced glycated hemoglobin (HbA1c: SMD, −0.27; 95% CI, −0.47 to −0.07), triglyceride (TG: SMD, −0.20; 95% CI, −0.34 to −0.06), body weight (SMD, −0.19; 95% CI, −0.36 to −0.03), and body mass index (BMI: SMD, −0.28; 95% CI, −0.42 to −0.14). Stricter carbohydrate restriction (<26% energy) further improved systolic/diastolic blood pressure, homeostatic model assessment insulin resistance index (HOMA-IR), HbA1c, TG, body weight, BMI, and waist circumference. Short-term interventions (<24 weeks) lowered HbA1c, TG, and BMI.

**Conclusion:**

This systematic review and meta-analysis found that LCDs are associated with improvements in cardiometabolic risk factors among patients with MASLD. Furthermore, short-term implementation of a strict carbohydrate-restricted dietary regimen may yield additional clinical benefits. Future research should prioritize: standardized nutrient assessment, enhanced adherence strategies, and cardiovascular endpoint trials.

**Systematic review registration:**

PROSPERO: CRD42024603432; https://www.crd.york.ac.uk/PROSPERO/view/CRD42024603432.

## 1 Introduction

Metabolic dysfunction-associated steatotic liver disease (MASLD), previously known as non-alcoholic fatty liver disease (NAFLD), represents the hepatic manifestation of metabolic syndrome and has reached pandemic proportions, with a global prevalence exceeding 30% (1). Recent studies indicate a significant increase in global MASLD prevalence: from 25.26% in 1990–2006 to 38.00% in 2016–2019. Geographically, prevalence peaks in Latin America (44.37%), followed by the Middle East and North Africa (36.53%), South Asia (33.83%), South-East Asia (33.07%), North America (31.20%), East Asia (29.71%), and Asia Pacific (28.02%), while Western Europe reports the lowest burden (25.10%) (2). The recent nomenclature shift to MASLD underscores its strong association with cardiometabolic dysregulation (3, 4), including obesity, insulin resistance, and dyslipidemia (4–7). The increased prevalence of MASLD is parallel to the increasing rates of obesity and type 2 diabetes (T2D) (8, 9). Approximately 10–30% of individuals with isolated steatosis progress to metabolic dysfunction-associated steatohepatitis (MASH) and advanced liver disease; however, concurrent T2D elevates this risk to 65% (10). Emerging data indicate that MASLD patients have an elevated risk of cardiovascular mortality, which now surpasses liver-related complications as the primary cause of death (11, 12). Additionally, MASLD elevates risks of extrahepatic conditions: chronic kidney disease (13), and several extrahepatic cancers (78% higher for uterine cancer, 38% higher for colorectal cancer, 2.5-fold for bladder cancer, and 2-fold for kidney cancer) (14). The economic burden of MASLD is substantial and growing. In 2016, direct annual healthcare costs in the USA reached $103 billion, exceeding the combined costs of Germany, France, and Italy (€27.7 billion) and the UK (£5.24 billion). Over the next decade, this burden is projected to rise to $1.005 trillion in the USA and €334 billion in Europe (15).

Despite its substantial clinical burden and the projected healthcare costs, therapeutic options are largely limited to lifestyle modifications, and the efficacy of pharmacological interventions remains unsatisfactory. Current pharmacological management of MASLD faces significant unmet needs. Among metabolic agents, glucagon-like peptide-1 receptor (GLP-1R) agonists (e.g., semaglutide) reduce hepatic fat through weight loss, but evidence for fibrosis improvement remains insufficient (16); peroxisome proliferator-activated receptor (PPAR) agonists (e.g., pioglitazone) improve histological features of steatohepatitis, yet adverse effects (weight gain, edema, potential heart failure) constrain clinical utility (17); sodium-dependent glucose transporters-2 (SGLT-2) inhibitors ameliorate glucolipid metabolism but lack controlled trials assessing histological endpoints. For liver-targeted therapies, resmetirom (a thyroid hormone receptor β agonist) is the only MASH-targeting drug with positive results from a registrational phase III clinical trial (18), though long-term safety (e.g., thyroid/gonadal effects) and hard endpoint benefits require further validation; farnesoid X receptor (FXR) agonists (e.g., obeticholic acid) failed approval due to hepatotoxicity and unfavorable risk–benefit profiles. In patients with cirrhosis or hepatocellular carcinoma, comprehensive strategies such as chemotherapy, resection, or transplantation are warranted. Collectively, limitations in histological response rates, safety profiles, and long-term outcomes underscore the imperative to explore adjunctive approaches for halting disease progression and mitigating cardiovascular risk. Nutritional interventions, such as low-carbohydrate diets (LCDs), intermittent energy restriction, and calorie-restricted diets, are considered beneficial for MASLD (19).

Among nutritional strategies, LCDs have garnered particular attention due to their dual potential to ameliorate both hepatic steatosis and associated metabolic derangements (20). Mechanistic studies in animal models demonstrate that LCDs reduce hepatic diacylglycerol (DAG) accumulation, shift ceramide synthesis toward beneficial very long-chain species, and upregulate mitochondrial fatty acid oxidation genes. These effects collectively suppress lipogenesis, exert anti-steatotic effects, and preserve insulin signaling (21). Clinical evidence further supports their efficacy: LCDs promote weight loss while simultaneously enhancing insulin sensitivity, preserving β-cell function, and optimizing glucometabolic parameters (22). Currently, a unified definition of LCDs is lacking. It is generally accepted that diets with carbohydrate intake <20 g/day (or <10% of total calories from carbohydrates) are considered ketogenic, <130 g/day (or <26% of total calories) are regarded as low carbohydrate, and diets with <45% of total calories from carbohydrates are classified as mild low carbohydrate diets (23, 24).

However, the paradigm of increased dietary fat consumption inherent to LCDs introduces critical clinical uncertainties. Controversies persist regarding the role of dietary fat in cardiometabolic health (25). This cardiovascular vulnerability raises concerns about LCD-induced alterations in lipid profiles, particularly elevated LDL-cholesterol levels observed in some trials (26, 27). Consequently, uncertainty regarding the cardiovascular impact of LCDs limits their use in MASLD patient populations. Given that international multidisciplinary expert consensus has endorsed LCDs as a key dietary strategy for MASLD management (19), investigating their impact on cardiovascular risk factors in MASLD patients holds clear theoretical and clinical significance.

To address this critical evidence gap, we conducted a systematic meta-analysis evaluating the cardiovascular safety profile of LCDs interventions in adults with MASLD. This study aims to support evidence-based, personalized dietary strategies that integrate hepatic recovery with cardiovascular protection in MASLD management.

## 2 Methods

### 2.1 Protocol and registration

The protocol of this review was published previously with a registration number CRD42024603432 on the International Prospective Register of Systematic Reviews (PROSPERO). This study was conducted and reported according to the Preferred Reporting Items for Systematic Reviews and Meta-Analyses (PRISMA) guidelines (Supplementary Table S1).

### 2.2 Search strategy

The type of study included in this review was randomized controlled trials (RCTs) with no language restrictions. PubMed, Cochrane Library databases, Web of Science, and Scopus were searched until March 19, 2025. The search included terms such as “non-alcoholic fatty liver disease,” “metabolic dysfunction-associated steatotic liver disease,” “metabolic dysfunction-associated fatty liver disease,” “diet, carbohydrate-restricted,” and “randomized controlled trial.” The detailed search strategy is provided in Supplementary Table S2. For details on the screening process, see Section 2.4.

### 2.3 Inclusion and exclusion criteria

The inclusion criteria were: (1) population: adult patients with MASLD; (2) intervention: LCDs defined as <45% total energy from carbohydrates, with duration ≥2 weeks; (3) comparison: any non-LCD dietary intervention, standard care, medication, or placebo; (4) outcomes: systolic blood pressure (SBP), diastolic blood pressure (DBP), fasting blood glucose (FBG), homeostatic model assessment insulin resistance index (HOMA-IR), glycated hemoglobin (HbA1c), low-density lipoprotein cholesterol (LDL-C), high-density lipoprotein cholesterol (HDL-C), triglyceride (TG), total cholesterol (TC), body weight, body mass index (BMI), waist circumference (WC), and waist-hip ratio (WHR); and (5) study design: studies with an RCT design. Exclusion criteria included duplicate literature, review articles, letters, case reports, conference proceedings, lack of data availability, non-randomized controlled trials, and animal studies.

### 2.4 Data extraction

Two authors (SP and SZ) were responsible for data extraction. Disagreements were resolved through discussion between reviewers or with a third reviewer (YG) when necessary. The following information was extracted: first author, publication year, age of the participants, study size, number of cases, intervention measures, duration of diet program, dietary intake assessment, and outcomes of interest (blood pressure, blood glucose, blood lipids, body composition indicators, etc.). When the amount of macronutrient intake was provided with g/day, we transformed the figures into percentages of total calorie intake (carbohydrate % of total energy = ( $\frac{\text{Carbohydrate}\;(g/\mathrm{day})\times 4}{\text{Total daily energy}\;(\text{kcal})}$ ) × 100%). The results were converted to a uniform scale when the studies measured outcomes in various ways (to convert blood glucose from mmol/L to mg/dL, multiply by 18.0; for LDL-C, HDL-C, and TC from mmol/L to mg/dL, multiply by 38.7; for TG from mmol/L to mg/dL, multiply by 88.6; to convert HbA1c from mmol/mol to %, divide by 10.929 and add 2.15).

### 2.5 Risk of bias assessment

Methodological quality was evaluated using the Cochrane Risk of bias assessment tool. The assessment covered seven domains: random sequence generation/allocation concealment (selection bias), blinding of participants and personnel (performance bias), blinding of outcome assessment (detection bias), incomplete outcome data (attrition bias), selective reporting (reporting bias), and other biases. Other biases were defined as follows: at least one significant risk of bias related to the specific study design, trial conduct, or context—for example: significant baseline imbalance between groups; conflicts of interest in funding or questionable research practices; inadequate total sample size (<20 participants); protocol deviations affecting validity (e.g., unplanned co-interventions); or any other problem threatening study integrity. Each section was rated on three levels: low risk, high risk, and unclear. The overall risk of bias was determined through consensus between the two investigators (JZ and YL), resolving any disagreements through discussion and consultation with a third researcher (JD). The risk of bias graph visualizes the distribution of bias across domains, while the risk of bias summary profiles individual study bias assessments.

### 2.6 Statistical analysis

Review Manager 5.4 was utilized to analyze the literature. Results were summarized using standard mean differences (SMD) with 95% confidence intervals (CI). In general, studies with *I*^2^ values of 25%, 50%, and 75% indicate low, moderate, and high levels of heterogeneity, respectively. Statistical significance was set at *p*-values <0.05 (two-tailed). Subgroup analyses were conducted based on dietary carbohydrate intake and intervention duration. A sensitivity analysis employing the leave-one-out method was performed to explore the source of heterogeneity. For meta-analyses comprising 10 or more studies, publication bias was evaluated using a dual-method approach: (1) visual assessment of asymmetry through funnel plot analysis and (2) statistical quantification using Egger’s regression test, with statistical significance set at *p* > 0.05 for the absence of publication bias.

## 3 Results

### 3.1 Search results and study selection

**Figure 1:**
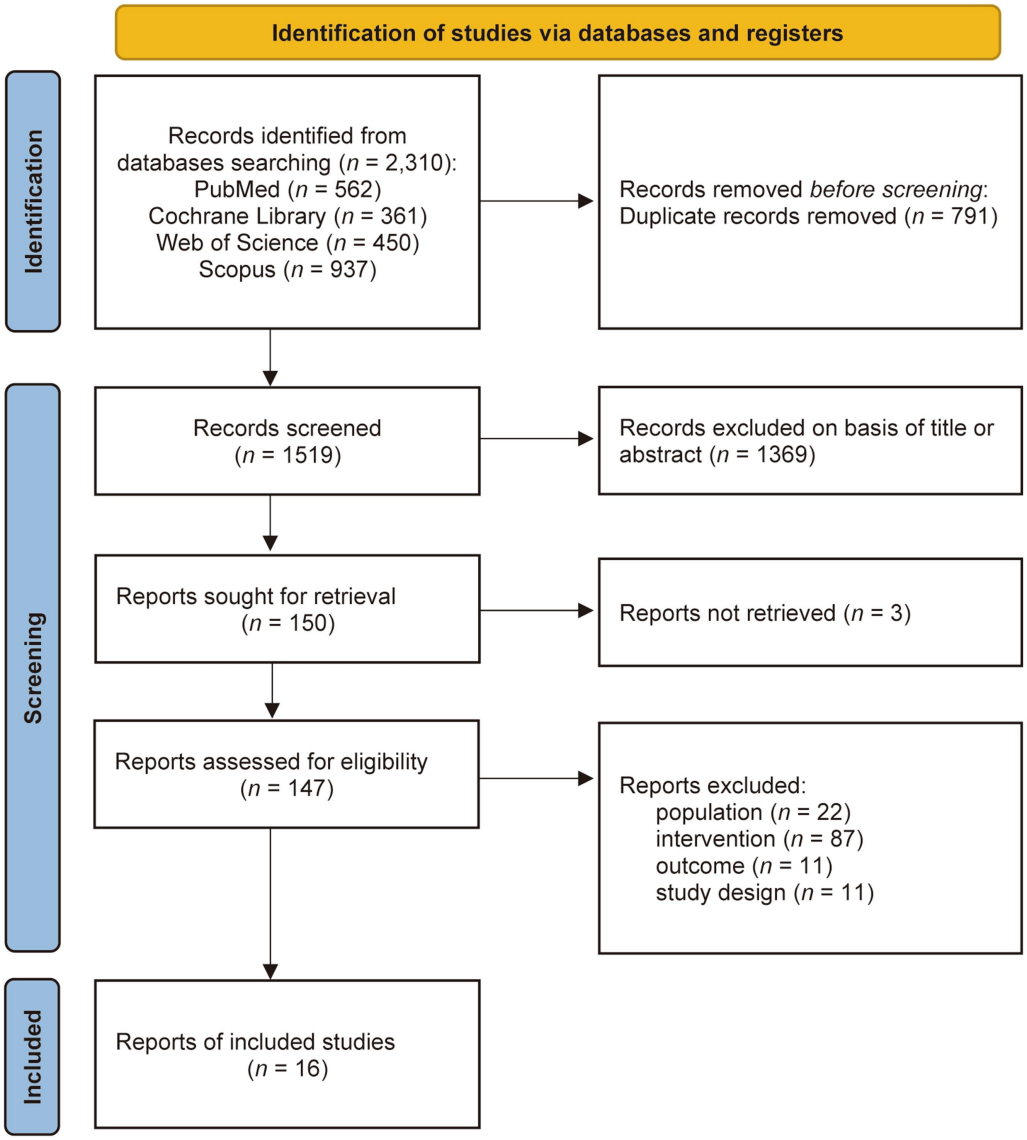
PRISMA flow diagram.

The results of the screening process are presented in Figure 1. As of March 19, 2025, 2,310 records were identified through systematic database searches. After deduplication, 1,519 records underwent title and abstract screening, resulting in 150 records for full-text review. Subsequently, 134 articles were excluded based on the following criteria: unretrievable publications (*n* = 3), non-compliant population characteristics (*n* = 22), non-conforming interventions (*n* = 87), ineligible outcome measures (*n* = 11), and non-RCT designs (*n* = 11). Ultimately, 16 studies met the inclusion criteria (28–43).

### 3.2 Study characteristics

Table 1 shows the main characteristics of the eligible studies. A total of 1,056 individuals with MASLD were included. Fifteen studies encompassed both males and females, whereas one study exclusively included males (31). Across these studies, the sample size varied from 18 to 226 participants, with a mean age ranging from 35 to 57 years. The studies were conducted in China (four studies, *n* = 465), Spain (three studies, *n* = 224), Australia (three studies, *n* = 114), Iran (two studies, *n* = 74), Sweden (one study, *n* = 74), Turkey (one study, *n* = 63), Thailand (one study, *n* = 24), and USA (one study, *n* = 18). Eleven (68.75%) of 16 studies reported missing participant outcome data (31, 33–41, 43), with 4 studies having an attrition rate exceeding 20% (33, 35, 37, 41).

**Table 1:** Characteristics of included studies.

Study	Country	Mean age (years: C/I)	*N* (C/I)	Carbohydrate intake	Comparator	Duration (weeks)	Dropout	Outcome
de Luis et al. (28)	Spain	45.5/46.8	15/13	CHO 38% Calorically restricted	Low-fat Calorically restricted	12	NA	SBP, DBP, HOMA-IR, HDL-C, TG, TC, body weight, BMI, WC, WHR
Arefhosseini et al. (29)	Iran	38.0/40.6	22/22	CHO 40% Calorically restricted	Low-calorie	6	NA	LDL-C, HDL-C, TG, TC, body weight, BMI
Browning et al. (30)	USA	47/42	9/9	CHO <20 g/day	Low-calorie	2	NA	FBG, TG, TC, body weight, BMI
Xu et al. (31)	China	35/37	27/31	CHO 35%	CHO 66%	6	10.3%	FBG, LDL-C, HDL-C, TG, TC, body weight, WC
Kani et al. (32)	Iran	45.6/49.3	15/15	CHO 45% Calorically restricted	Low-calorie	8	NA	SBP, DBP, FBG, LDL-C, HDL-C, TG, TC, body weight, BMI
Croci et al. (33)	Australia	51.8/45.5	13/8	CHO 40%	Exercise training	24	23.8%	SBP, DBP, FBG, LDL-C, HDL-C, TG, BMI, WC
Properzi et al. (34)	Australia	53/51	25/26	CHO 40%	Low-fat	12	5.9%	SBP, DBP, FBG, HOMA-IR, HbA1c, LDL-C, HDL-C, TG, TC, BMI, WC
Marin-Alejandre et al. (35)	Spain	51.1/49.2	48/50	CHO 40–45% Calorically restricted	Balanced diet 3–5 meals/day Calorically restricted	24	22.4%	SBP, DBP, FBG, HOMA-IR, LDL-C, HDL-C, TG, TC, body weight, BMI, WC
Holmer et al. (36)	Sweden	57/56	49/25	CHO 5–10% Calorically restricted	Intermittent calorie restriction Standard of care (two control arms)	12	13.5%	SBP, DBP, HOMA-IR, HbA1c, LDL-C, HDL-C, TG, TC, body weight, BMI, WHR
Marin-Alejandre et al. 2021 (37)	Spain	51.1/49.2	48/50	CHO 40–45% Calorically restricted	Balanced diet 3–5 meals/day Calorically restricted	96	40.8%	SBP, DBP, FBG, HOMA-IR, HbA1c, LDL-C, HDL-C, TG, TC, body weight, BMI, WC
George et al. (38)	Australia	52.1/52.6	23/19	CHO 33%	Low-fat	12	7.1%	SBP, DBP, FBG, HOMA-IR, LDL-C, HDL-C, TG, TC, body weight, BMI, WC
Sun et al. (39)	China	38.9/39.8	33/30	CHO 20–25% Calorically restricted	Balanced diet Calorically restricted	12	6.3%	SBP, DBP, FBG, HOMA-IR, HbA1c, LDL-C, HDL-C, TG, TC, BMI, WC
Feng et al. (40)	China	45.5/41.0	79/39	CHO 40–50%	Calorically restricted and exercise 120 mg of orlistat 3 times/day (two control arms)	24	12.7%	SBP, DBP, FBG, HOMA-IR, LDL-C, HDL-C, TG, TC, body weight, BMI, WC, WHR
Liu et al. (41)	China	36.7/36.7 (media)	115/111	CHO 20–25% Calorically restricted	Balanced diet Calorically restricted	12	25.2%	SBP, DBP, FBG, HOMA-IR, HbA1c, LDL-C, HDL-C, TG, TC, BMI
Uluçay Kestane and Baş (42)	Turkey	39.1/39.7	42/21	CHO ≤35% Mediterranean diet	Typical Mediterranean diet Low-fat Mediterranean diet (two control arms)	8	NA	HOMA-IR, body weight, BMI, WC, WHR
Chirapongsathorn et al. (43)	Thailand	40.2/37.4	12/12	CHO 5% Calorically restricted	DASH diet Calorically restricted	8	8.3%	SBP, DBP, FBG, LDL-C, HDL-C, TG, TC, body weight, BMI, WC

C/I, control/intervention; *N*, sample size; CHO, carbohydrate; SBP, systolic blood pressure; DBP, diastolic blood pressure; FBG, fasting blood glucose; HOMA-IR, homeostatic model assessment insulin resistance index; HbA1c, glycated hemoglobin; LDL-C, low-density lipoprotein cholesterol; HDL-C, high-density lipoprotein cholesterol; TG, triglyceride; TC, total cholesterol; BMI, body mass index; WC, waist circumference; WHR, waist-hip ratio.

Of the 16 RCTs, depending on the carbohydrate content of the diet, 2 studies were <10% (30, 43), 3 studies were <26% (36, 39, 41), and 11 studies were ≥26% (28, 29, 31–35, 37, 38, 40, 42). Intervention durations ranged from 2 to 96 weeks, with an overall median of 12 weeks. Of these, 12 studies (62.5%) implemented short-term interventions (<24 weeks) (28–32, 34, 36, 38, 39, 41–43), and 4 studies (37.5%) longer-term interventions (≥24 weeks) (33, 35, 37, 40). Control interventions include regular carbohydrate diet (31, 35, 37, 39, 41), low-fat diet (28, 34, 38, 42), low calorie diet (29, 30, 32), intermittent calorie restriction (36), exercise training (33, 40), Mediterranean diet (42), Dietary Approaches to Stop Hypertension (DASH) diet (43), orlistat (40), and standard of care (36). Supplementary Table S3 summarizes available fat composition data from the included studies, demonstrating that only 7 of 16 trials (43.75%) quantified fatty acid subtypes; of these, two (30, 38) reported saturated fatty acids (SFA) levels exceeding 10%.

### 3.3 Risk of bias

**Figure 2:**
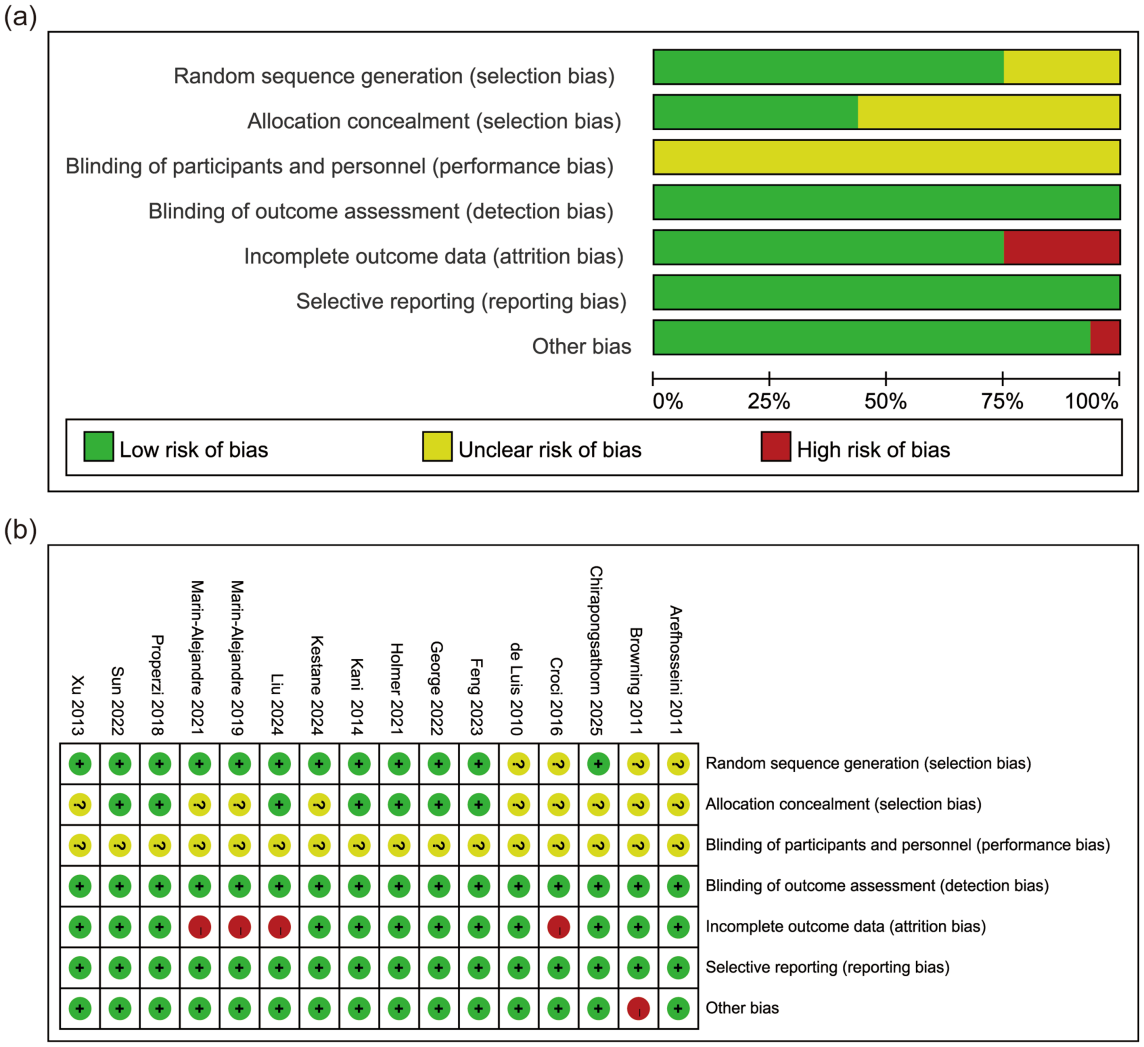
Risk of bias assessment for included studies. **(a)** Risk of bias graph. **(b)** Risk of bias summary.

Figure 2 shows the Risk of Bias assessments of the included trials. Four RCTs had no information on random sequence generation (28–30, 33) and nine had no information on allocation concealment (28–31, 33, 35, 37, 42, 43). The risk level of performance bias was considered unclear in all studies, as blinding of dietary and exercise interventions was not feasible. The attrition bias of four studies (33, 35, 37, 41) was regarded as high risk as the dropout rate exceeded 20%. High attrition rates could inflate efficacy estimates, as participants with suboptimal responses or poor adherence are more likely to withdraw. Other bias was rated as high risk in one study (30). This designation resulted from its inadequate sample size (intervention group: *n* = 9; control group: *n* = 9), which falls below our pre-specified threshold of 20 participants. This limitation may increase susceptibility to random error, and may reduce statistical power to detect true effects.

### 3.4 Outcomes of the studies

#### 3.4.1 Blood pressure

**Figure 3:**
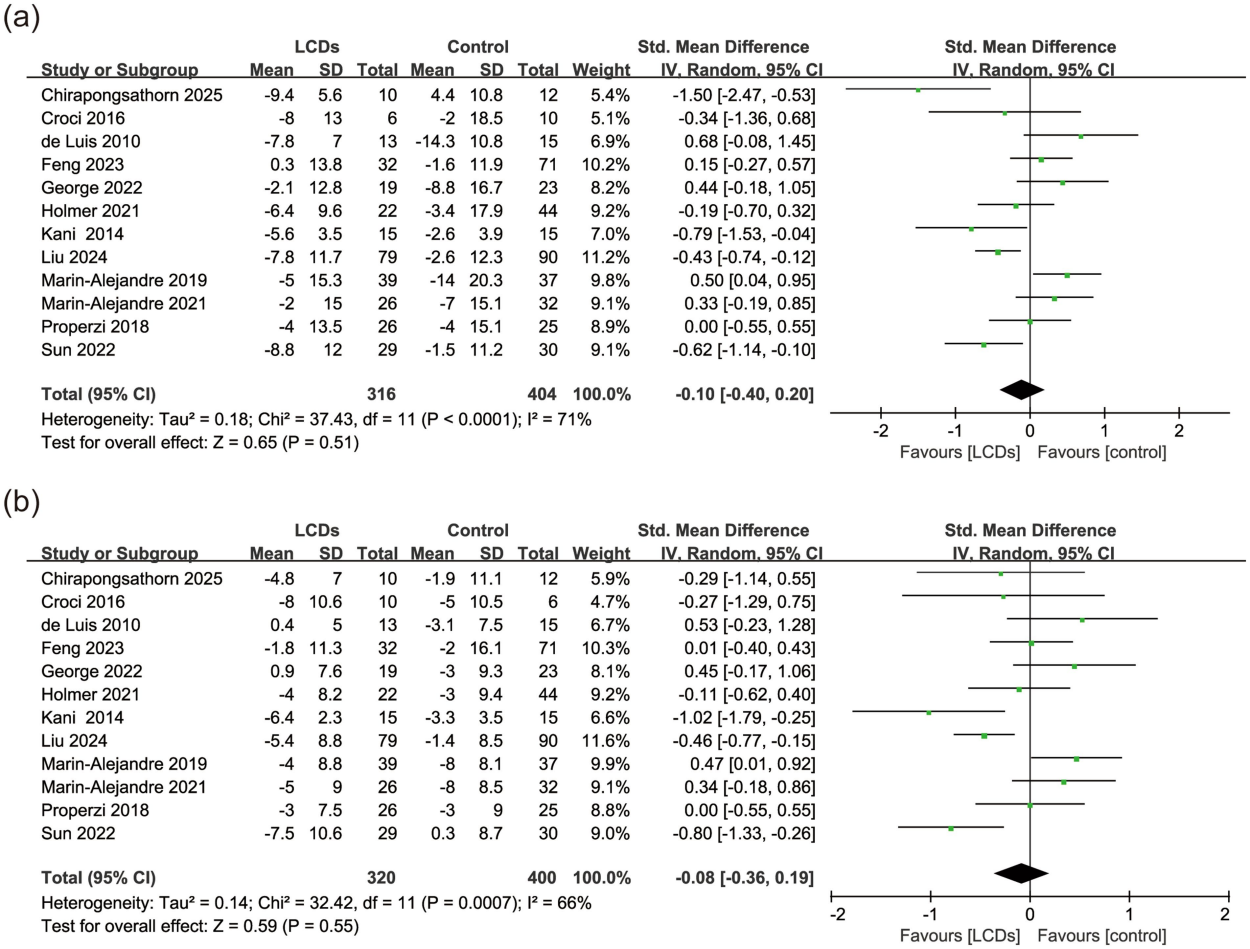
The effect of LCDs on **(a)** SBP and **(b)** DBP.

Twelve (75.0%) of 16 studies reported the effects on blood pressure (28, 32–41, 43). As the highest-weight contributor to both SBP and DBP analyses, Liu et al. (41) achieved substantial reductions (−7.8/−5.4 mmHg) through intensive dietitian-led counseling—a key adherence mediator despite 25.2% COVID-19 dropout. In within-group analyses, the LCDs arms showed SBP reductions in 11 studies (mean change range: −2.0 to −9.4 mmHg), but only 4 trials demonstrated statistically significant between-group differences vs. controls. Similarly, 10 studies reported DBP reductions in LCD groups (range: −1.8 to −8.0 mmHg), with just 3 showing significant between-group effects. Despite these numerical improvements, meta-analysis revealed no significant difference in SBP (SMD, −0.10; 95% CI, −0.40 to 0.20, *p* = 0.51, *I*^2^ = 71%, *n* = 720), or DBP (SMD, −0.08; 95% CI, −0.36 to 0.19, *p* = 0.55, *I*^2^ = 66%, *n* = 720) between the LCDs and control groups (Figure 3). However, the high heterogeneity could not be further reduced using the leave-one-out method in both systolic and diastolic blood pressure. Subgroup analysis indicated that implementation of a strict LCD intervention (<26%) resulted in significant reductions in both SBP (SMD, −0.53; 95% CI, −0.89 to −0.17, *p* = 0.004, *I*^2^ = 49%, *n* = 316) and DBP (SMD, −0.44; 95% CI, −0.69 to −0.18, *p* < 0.001, *I*^2^ = 13%, *n* = 316) (Supplementary Figure S1). The control group demonstrated a statistically superior reduction in SBP compared to the LCD group during long-term intervention (SMD, 0.27; 95% CI, 0.02 to 0.53, *p* = 0.04, *I*^2^ = 0%, *n* = 253). No statistical significance was observed in the subgroup analyses of other outcomes (Supplementary Figure S5).

#### 3.4.2 Glycemic control

**Figure 4:**
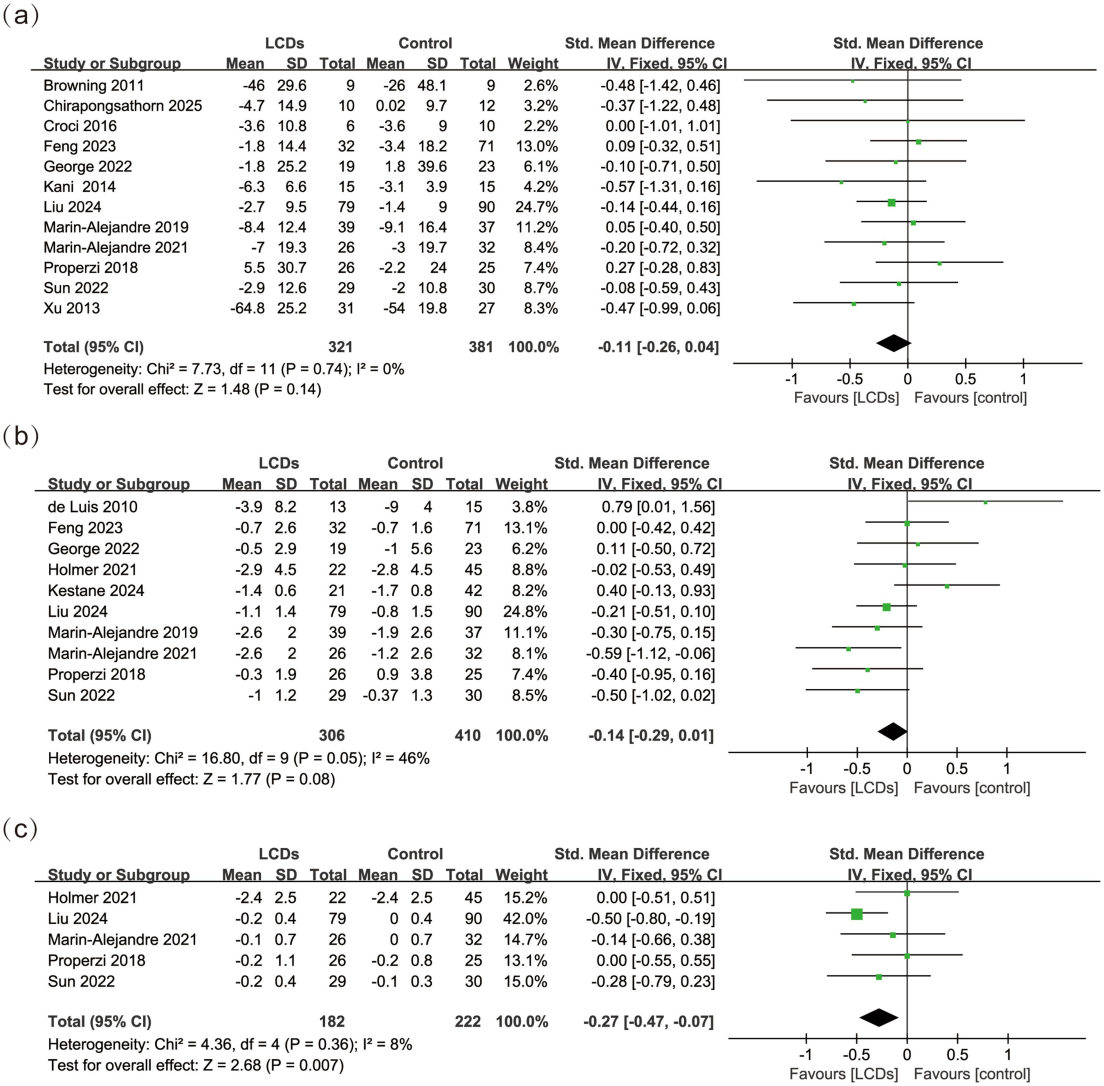
The effect of LCDs on **(a)** FBG, **(b)** HOMA-IR, and **(c)** HbA1c.

Fifteen (93.75%) of 16 studies reported the effects on glycemic control (28, 30–43). Analysis of the collected data showed significantly improved HbA1c (SMD, −0.27; 95% CI, −0.47 to −0.07, *p* = 0.007, *I*^2^ = 8%, *n* = 404), but no significant effect of LCDs on FBG (SMD, −0.11; 95% CI, −0.26 to 0.04, *p* = 0.14, *I*^2^ = 0%, *n* = 702), or HOMA-IR (SMD, −0.14; 95% CI, −0.29 to 0.01, *p* = 0.08, *I*^2^ = 46%, *n* = 716) (Figure 4). Subgroup analysis demonstrated that both strict carbohydrate restriction (SMD, −0.35; 95% CI, −0.58 to −0.11, *p* = 0.004, *I*^2^ = 28%, *n* = 295) and shorter intervention duration (SMD, −0.29; 95% CI, −0.51 to −0.08, *p* = 0.007, *I*^2^ = 26%, *n* = 346) were associated with significant reductions in HbA1c levels (Supplementary Figures S2, S6). Notably, the strict LCD subgroup (<26%) comprised three studies with carbohydrate intakes of 12.8%, 19.8%, and 24.6% of total energy, respectively (36, 39, 41); while the short-term intervention subgroup (<24 weeks) consisted of four studies all implementing 12-week interventions (34, 36, 39, 41). No statistical significance was observed in the subgroup analyses of other outcomes.

#### 3.4.3 Lipid profiles

**Figure 5:**
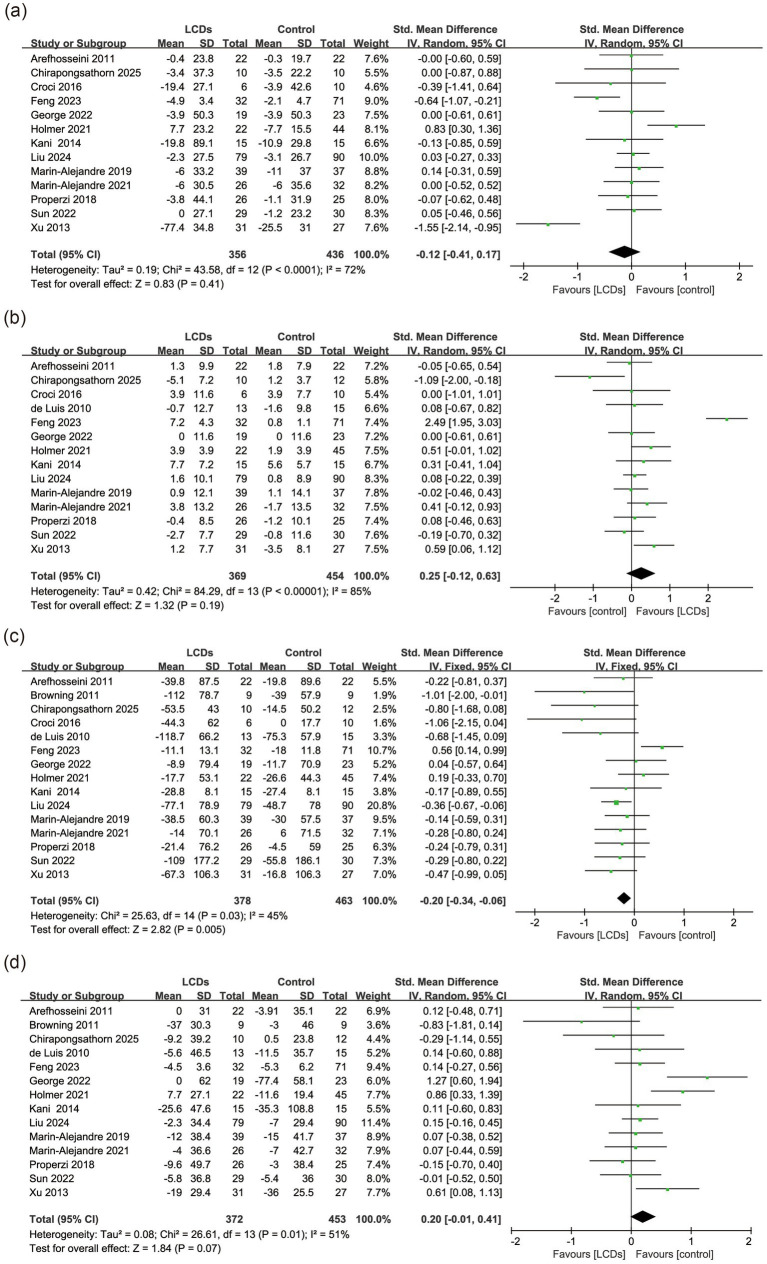
The effect of LCDs on **(a)** LDL-C, **(b)** HDL-C, **(c)** TG, and **(d)** TC.

Fifteen (93.75%) of 16 studies reported the effects on lipid profiles (28–41, 43), specifically 15 on TG, 14 on TC, 13 on LDL-C, and 14 on HDL-C. In contrast to other trials, Xu’s et al. (31) trial exclusively enrolled male participants. George et al. (38) prescribed 44% total energy from fat in the LCD group, with >50% derived from monounsaturated fatty acids (MUFA). Post-intervention dietary assessment demonstrated fatty acid distribution in the LCD group: saturated fatty acids (SFA) 10.8%, MUFA 19.0%, polyunsaturated fatty acids (PUFA) 7.7%. Feng et al. (40) included a control arm comprising pharmacotherapy (orlistat 120 mg three times daily). The LCDs were associated with lower TG levels (SMD, −0.20; 95% CI, −0.34 to −0.06, *p* = 0.005, *I*^2^ = 45%, *n* = 841). No significant difference was observed in LDL-C (SMD, −0.12; 95% CI, −0.41 to 0.17, *p* = 0.41, *I*^2^ = 72%, *n* = 792), HDL-C (SMD, 0.25; 95% CI, −0.12 to 0.63, *p* = 0.19, *I*^2^ = 85%, *n* = 823) and or TC (SMD, 0.20; 95% CI, −0.01 to 0.41, *p* = 0.07, *I*^2^ = 51%, *n* = 825), between the LCDs and control groups (Figure 5). Sensitivity analysis revealed that the exclusion of Xu’s et al. (31) study markedly reduced heterogeneity in LDL-C analysis (*I*^2^ = 42%), though the overall effect remained nonsignificant (*p* = 0.99). Similarly, the exclusion of George’s et al. (38) study substantially lowered heterogeneity for TC analysis (*I*^2^ = 27%), yet the pooled effect did not reach statistical significance (*p* = 0.11). Finally, the exclusion of Feng’s et al. (40) study markedly reduced heterogeneity in HDL-C analysis (*I*^2^ = 24%), though the overall effect remained nonsignificant (*p* = 0.22). Subgroup analysis demonstrated that both strict carbohydrate restriction (SMD, −0.30; 95% CI, −0.52 to −0.09, *p* = 0.007, *I*^2^ = 41%, *n* = 335) and shorter intervention duration (SMD, −0.29; 95% CI, −0.46 to −0.13, *p* < 0.001, *I*^2^ = 0%, *n* = 588) were associated with significant reductions in TG levels (Supplementary Figures S3, S7). No statistical significance was observed in the subgroup analyses of other outcomes.

#### 3.4.4 Anthropometric indicators

**Figure 6:**
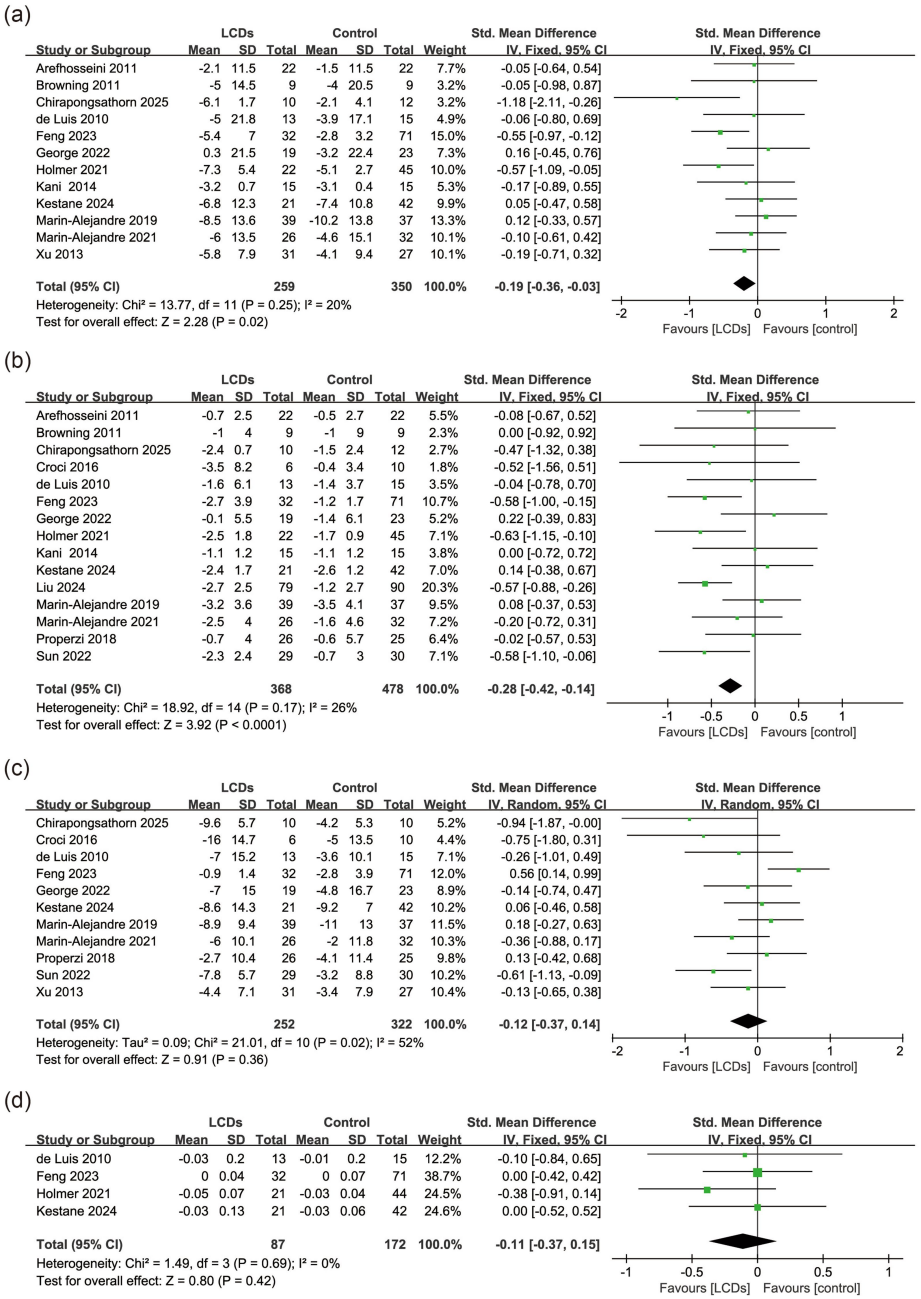
The effect of LCDs on **(a)** body weight, **(b)** BMI, **(c)** WC, and **(d)** WHR.

All studies reported the effects on anthropometric indicators (28–43). Feng et al. (40) had the highest weight in the pooled analyses of body weight (15.0%), waist circumference (12.0%), and WHR (38.7%). Although specific fatty acid profiles were not quantified, their intervention implemented a diet characterized by richness in monounsaturated and omega-3 fatty acids, high vegetable content, and abundant soluble fiber. Liu et al. (41) accounted for the greatest weight in BMI analysis (20.3%), explicitly recommending PUFA as the primary fat source during intervention. The LCDs significantly decreased body weight (SMD, −0.19; 95% CI, −0.36 to −0.03, *p* = 0.02, *I*^2^ = 20%, *n* = 609) and BMI (SMD, −0.28; 95% CI, −0.42 to −0.14, *p* < 0.001, *I*^2^ = 26%, *n* = 846). However, LCDs did not significantly reduce waist circumference (SMD, −0.12; 95% CI, −0.37 to 0.14, *p* = 0.36, *I*^2^ = 52%, *n* = 574) or WHR (SMD, −0.11; 95% CI, −0.37 to 0.15, *p* = 0.42, *I*^2^ = 0%, *n* = 259) (Figure 6). Sensitivity analysis revealed that the exclusion of the study (40) markedly reduced heterogeneity in waist circumference analysis (*I*^2^ = 20%), though the overall effect remained nonsignificant (*p* = 0.08). Subgroup analysis demonstrated that lower carbohydrate intake (<26%) significantly reduced body weight (SMD, −0.59; 95% CI, −1.00 to −0.18, *p* = 0.004, *I*^2^ = 31%, *n* = 107), BMI (SMD, −0.54; 95% CI, −0.77 to −0.32, *p* < 0.001, *I*^2^ = 0%, *n* = 335), and waist circumference (SMD, −0.69; 95% CI, −1.15 to −0.23, *p* = 0.003, *I*^2^ = 0%, *n* = 79). However, only one study (36) reported WHR outcomes (SMD, −0.38; 95% CI, −0.91 to 0.14, *p* = 0.15, *n* = 65), showing no significant reduction. Pooled analysis was unfeasible given single-study data availability (Supplementary Figure S4d). A statistically significant reduction in BMI was consistently observed in both short-term (<24 weeks) and long-term interventions (Supplementary Figures S4, S8). No statistical significance was observed in the subgroup analyses of other outcomes.

### 3.5 Publication bias

The funnel plot and Egger’s test indicated no significant publication bias in SBP (*p* = 0.867), DBP (*p* = 0.710), FBG (*p* = 0.312), HOMA-IR (*p* = 0.3226), LDL-C (*p* = 0.684), HDL-C (*p* = 0.863), TG (*p* = 0.113), TC (*p* = 0.784), body weight (*p* = 0.737), and BMI (*p* = 0.160). Potential publication bias was observed in waist circumference (*p* = 0.019), but trim-and-fill analysis revealed no missing studies requiring imputation (Supplementary Figures S9a–k). Publication bias was not assessed for HbA1c and WHR (<10 trial comparisons).

## 4 Discussion

This meta-analysis demonstrates that LCDs significantly improve key cardiometabolic parameters among patients with MASLD, including reductions in HbA1c, TG, body weight, and BMI. While the pooled analysis showed no statistically significant change in LDL-C (SMD, −0.12; 95% CI, −0.41 to 0.17, *p* = 0.41), the high heterogeneity (*I*^2^ = 72%) indicates substantial inconsistency between studies. Similarly, the nonsignificant results for HDL-C (*I*^2^ = 85%) and TC (*I*^2^ = 51%) must be interpreted with caution. Although LCDs were defined broadly as <45% carbohydrate energy, the included trials encompassed three subtypes: ketogenic (<10%; *n* = 2), strict low-carbohydrate (<26%; *n* = 3), and mild low-carbohydrate (26–45%; *n* = 11) diets. Importantly, subgroup analyses using the three-tier classification were precluded for 9 of 13 outcomes (SBP, DBP, HOMA-IR, HbA1c, LDL-C, HDL-C, body weight, WC, WHR) due to insufficient studies (<2 trials per subgroup). This limitation necessitated our dichotomous approach (<26% vs. ≥26% carbohydrate energy) to ensure statistical robustness. Moreover, stricter carbohydrate restriction (<26% total energy) was associated with additional clinical benefits, including significant improvements in blood pressure (systolic/diastolic), insulin sensitivity (HOMA-IR), and reductions in waist circumference. However, the lack of WHR data in this subgroup represents a critical evidence gap. Given that WHR more accurately reflects central adiposity distribution than waist circumference alone, future trials should prioritize standardized WHR measurement, especially when evaluating interventions targeting visceral fat reduction. Short-term LCDs interventions (<24 weeks) were particularly effective for glycemic control and lipid profile improvement. Our findings suggest that while low carbohydrate diets may be effective in reducing body weight and improving metabolic parameters in MASLD patients, their impact on cardiovascular risk factors is more complex and may vary depending on various factors, such as the duration and intensity of the diet.

Improving insulin resistance—a central pathophysiological link between MASLD and cardiovascular disease—is a cornerstone of LCDs efficacy (6, 44). Consistent with prior evidence (22), our findings demonstrate that LCDs significantly reduce insulin resistance. By reducing postprandial glycemic excursions and insulin secretion, carbohydrate restriction attenuates hepatic *de novo* lipogenesis (DNL) via suppression of carbohydrate-responsive element-binding protein (ChREBP) (45), while enhancing peripheral glucose uptake through AMP-activated protein kinase (AMPK)-mediated GLUT4 translocation (46). This dual mechanism not only reduces HOMA-IR but also disrupts the vicious cycle of ectopic lipid accumulation of MASLD. Furthermore, LCDs-induced reductions in dietary fructose intake mitigate hepatic lipid accumulation by downregulating lipogenic enzymes such as sterol regulatory element-binding protein 1c (SREBP-1c), thereby suppressing fatty acid synthesis and promoting mitochondrial β-oxidation (47).

The effects of LCDs on lipid profiles remain controversial (48). Studies in non-MASLD populations have reported elevated TC, LDL-C, and HDL-C levels with LCD interventions (49). However, our analysis of MASLD patients demonstrated TG was significantly reduced, while TC, LDL-C, and HDL-C remained unchanged. This divergence may reflect that MASLD patients characterized by insulin resistance, likely derive greater benefits from LCD-induced suppression of *de novo* lipogenesis and enhanced fatty acid oxidation.

Prolonged adherence may unmask the adverse effects of suboptimal fat composition. LCDs shift energy substrate utilization to fats, promoting adipocyte lipolysis (50), but excessive dietary fat intake is associated with an increased risk of CVD (51). Among dietary factors, saturated fatty acids exert the most significant influence on blood lipids (52). Replacing saturated fats with unsaturated fats not only attenuates LDL-C but also improves insulin sensitivity (53). Moreover, further research suggests that insulin resistance may confer a higher cardiovascular risk compared to LDL cholesterol (54). So to some extent, LCDs may provide significant benefits, including weight loss, increased insulin sensitivity, and a potentially greater reduction in cardiovascular risk (55). Given the critical roles of LDL-C and TC in cardiovascular risk (56, 57), LCDs that reduce carbohydrates and unsaturated fatty acids should be prioritized in MASLD management.

LCDs may lower blood pressure through multiple mechanisms, including reduced insulin levels, modulation of vascular and neuroendocrine systems, and weight loss. By limiting carbohydrate intake, LCDs decrease insulin-mediated sodium reabsorption in the kidneys, reducing sodium and water retention (58). Additionally, LCDs modulate vascular endothelial function, the hypothalamic-pituitary-adrenal (HPA) axis, the sympathetic nervous system (SNS), and the renin-angiotensin-aldosterone (RAA) system, all of which play critical roles in BP control (59–61). Weight loss, a common outcome of LCDs, further contributes to BP reduction through caloric restriction and improved body composition (62).

Consistent with previous studies, LCDs’ long-term efficacy appears less satisfactory. One potential explanation is that reduced glycogen stores associated with LCDs adoption may lead to decreased physical activity and increased fatigue, ultimately resulting in diminished energy expenditure (63). Sustained adherence to LCDs may pose significant challenges, and the therapeutic benefits of dietary interventions are largely constrained by participant compliance. To address these limitations, future long-term prospective studies should investigate the sustained effects of LCDs on cardiovascular outcomes.

Our findings may provide reassurance to healthcare providers and patients considering the use of LCDs for the management of MASLD, as not all high-fat diets necessarily result in a substantial increase in CVD risks. These findings advocate a phased clinical approach to LCDs implementation in MASLD. Short-term interventions with strict carbohydrate restriction may rapidly improve insulin sensitivity and dyslipidemia by leveraging ketosis-driven appetite suppression and increased energy expenditure (64, 65). Prolonged adherence should prioritize unsaturated fats and high-fiber foods to mitigate lipid risks while sustaining metabolic benefits.

Some limitations should be considered. First, participants were unlikely to be blinded due to the study design, and self-reported dietary data could be affected by recall inaccuracies. Second, evaluation of WHR was feasible in only one study within the strict LCD subgroup, preventing meaningful assessment of its clinical utility. Third, heterogeneity in dietary fat composition across trials (e.g., saturated vs. unsaturated fats) obscured whether observed effects stemmed from carbohydrate restriction or fat quality changes. Fourth, the high attrition rates (>20%) in 25% of included trials may introduce selection bias. Participants with suboptimal responses or poor dietary adherence are more likely to withdraw, potentially inflating efficacy estimates. Fifth, while a three-tier classification (ketogenic/strict/mild) is theoretically preferable, sparse data for ketogenic diets (<10%; only 2 trials) limited subgroup comparisons. Finally, short intervention durations precluded assessment of long-term cardiovascular outcomes.

Future research efforts would benefit from focusing on the following critical aspects: (1) Standardizing nutrient quantification in LCDs to include daily carbohydrate intake in absolute grams and percentage of total energy, alongside detailed characterization of fat subtypes—specifically ratios of saturated, monounsaturated, and polyunsaturated fatty acids with primary sources identified (e.g., coconut oil, olive oil). (2) Implementing real-time dietary monitoring via validated digital diaries to reduce recall bias, and introducing interventions (e.g., personalized counseling, peer support networks) to improve long-term compliance. (3) Conducting long-term trials with definitive cardiovascular endpoints (e.g., major adverse cardiovascular events, cardiovascular mortality, carotid plaque progression) to evaluate the cardiovascular efficacy and safety of LCDs.

## 5 Conclusion

In patients with MASLD, LCDs demonstrate clinically meaningful improvements in insulin sensitivity, adiposity, and dyslipidemia, particularly when carbohydrate intake is restricted below 26% of total energy. To reconcile hepatic and cardiovascular health, it is necessary to emphasize carbohydrate restriction. Future research should prioritize: standardized nutrient assessment, enhanced adherence strategies, and cardiovascular endpoint trials.

## Data Availability

The original contributions presented in the study are included in the article/Supplementary material, further inquiries can be directed to the corresponding author.
