# Supplementary material for: Low-carbohydrate diets reduce cardiovascular risk factor levels in patients with metabolic dysfunction-associated steatotic liver disease: a systematic review and meta-analysis of randomized controlled trials
Source: Front Nutr. 2025 Aug 26;12:1626352. doi: 10.3389/fnut.2025.1626352 (PMC12417190; doi:10.3389/fnut.2025.1626352)
Supplement: Supplementary file 1 [file Table_1.docx]

Supplementary material

[PRISMA 2020 Checklist 2](#_Toc205924799)

[Search strategy 6](#_Toc205924800)

[Subgroup analysis stratified by dietary carbohydrate intake 9](#_Toc205924801)

[Subgroup analysis stratified by intervention duration 15](#_Toc205924802)

[Funnel plots 21](#_Toc205924803)

[Dietary Fat Composition 27](#_Toc205924804)

# PRISMA 2020 Checklist

Table S1 PRISMA 2020 Checklist

| **Section and Topic** | **Item #** | **Checklist item** | **Location where item is reported** |
| --- | --- | --- | --- |
| **TITLE** | | |  |
| Title | 1 | Identify the report as a systematic review. | Title |
| **ABSTRACT** | | |  |
| Abstract | 2 | See the PRISMA 2020 for Abstracts checklist. | Abstract |
| **INTRODUCTION** | | |  |
| Rationale | 3 | Describe the rationale for the review in the context of existing knowledge. | Introduction |
| Objectives | 4 | Provide an explicit statement of the objective(s) or question(s) the review addresses. | Introduction |
| **METHODS** | | |  |
| Eligibility criteria | 5 | Specify the inclusion and exclusion criteria for the review and how studies were grouped for the syntheses. | Methods: Inclusion and Exclusion Criteria |
| Information sources | 6 | Specify all databases, registers, websites, organisations, reference lists and other sources searched or consulted to identify studies. Specify the date when each source was last searched or consulted. | Methods: Protocol and Registration; Search Strategy |
| Search strategy | 7 | Present the full search strategies for all databases, registers and websites, including any filters and limits used. | Methods: Search Strategy |
| Selection process | 8 | Specify the methods used to decide whether a study met the inclusion criteria of the review, including how many reviewers screened each record and each report retrieved, whether they worked independently, and if applicable, details of automation tools used in the process. | Methods: Data extraction |
| Data collection process | 9 | Specify the methods used to collect data from reports, including how many reviewers collected data from each report, whether they worked independently, any processes for obtaining or confirming data from study investigators, and if applicable, details of automation tools used in the process. | Methods: Data extraction |
| Data items | 10a | List and define all outcomes for which data were sought. Specify whether all results that were compatible with each outcome domain in each study were sought (e.g. for all measures, time points, analyses), and if not, the methods used to decide which results to collect. | Methods: Data extraction |
|  | 10b | List and define all other variables for which data were sought (e.g. participant and intervention characteristics, funding sources). Describe any assumptions made about any missing or unclear information. | Methods: Data extraction |
| Study risk of bias assessment | 11 | Specify the methods used to assess risk of bias in the included studies, including details of the tool(s) used, how many reviewers assessed each study and whether they worked independently, and if applicable, details of automation tools used in the process. | Methods: Risk of Bias Assessment |
| Effect measures | 12 | Specify for each outcome the effect measure(s) (e.g. risk ratio, mean difference) used in the synthesis or presentation of results. | Methods: Statistical Analysis |
| Synthesis methods | 13a | Describe the processes used to decide which studies were eligible for each synthesis (e.g. tabulating the study intervention characteristics and comparing against the planned groups for each synthesis (item #5)). | Methods: Data extraction |
|  | 13b | Describe any methods required to prepare the data for presentation or synthesis, such as handling of missing summary statistics, or data conversions. | Methods: Data extraction |
|  | 13c | Describe any methods used to tabulate or visually display results of individual studies and syntheses. | Methods: Statistical Analysis |
|  | 13d | Describe any methods used to synthesize results and provide a rationale for the choice(s). If meta-analysis was performed, describe the model(s), method(s) to identify the presence and extent of statistical heterogeneity, and software package(s) used. | Methods: Statistical Analysis |
|  | 13e | Describe any methods used to explore possible causes of heterogeneity among study results (e.g. subgroup analysis, meta-regression). | Methods: Statistical Analysis |
|  | 13f | Describe any sensitivity analyses conducted to assess robustness of the synthesized results. | Methods: Statistical Analysis |
| Reporting bias assessment | 14 | Describe any methods used to assess risk of bias due to missing results in a synthesis (arising from reporting biases). | Methods: Risk of Bias Assessment |
| Certainty assessment | 15 | Describe any methods used to assess certainty (or confidence) in the body of evidence for an outcome. | Methods: Risk of Bias Assessment |
| **RESULTS** | | |  |
| Study selection | 16a | Describe the results of the search and selection process, from the number of records identified in the search to the number of studies included in the review, ideally using a flow diagram. | Results: Search Results and Study Selection |
|  | 16b | Cite studies that might appear to meet the inclusion criteria, but which were excluded, and explain why they were excluded. | Results: Search Results and Study Selection |
| Study characteristics | 17 | Cite each included study and present its characteristics. | Results: Study Characteristics |
| Risk of bias in studies | 18 | Present assessments of risk of bias for each included study. | Results: Risk of bias |
| Results of individual studies | 19 | For all outcomes, present, for each study: (a) summary statistics for each group (where appropriate) and (b) an effect estimate and its precision (e.g. confidence/credible interval), ideally using structured tables or plots. | Results: Outcomes of the Studies |
| Results of syntheses | 20a | For each synthesis, briefly summarise the characteristics and risk of bias among contributing studies. | Results: Outcomes of the Studies |
|  | 20b | Present results of all statistical syntheses conducted. If meta-analysis was done, present for each the summary estimate and its precision (e.g. confidence/credible interval) and measures of statistical heterogeneity. If comparing groups, describe the direction of the effect. | Results: Outcomes of the Studies |
|  | 20c | Present results of all investigations of possible causes of heterogeneity among study results. | Results: Outcomes of the Studies |
|  | 20d | Present results of all sensitivity analyses conducted to assess the robustness of the synthesized results. | Results: Outcomes of the Studies |
| Reporting biases | 21 | Present assessments of risk of bias due to missing results (arising from reporting biases) for each synthesis assessed. | Results: Outcomes of the Studies |
| Certainty of evidence | 22 | Present assessments of certainty (or confidence) in the body of evidence for each outcome assessed. | Results: Outcomes of the Studies |
| **DISCUSSION** | | |  |
| Discussion | 23a | Provide a general interpretation of the results in the context of other evidence. | Discussion |
|  | 23b | Discuss any limitations of the evidence included in the review. | Discussion |
|  | 23c | Discuss any limitations of the review processes used. | Discussion |
|  | 23d | Discuss implications of the results for practice, policy, and future research. | Discussion |
| **OTHER INFORMATION** | | |  |
| Registration and protocol | 24a | Provide registration information for the review, including register name and registration number, or state that the review was not registered. | Methods: Protocol and Registration |
|  | 24b | Indicate where the review protocol can be accessed, or state that a protocol was not prepared. | NA |
|  | 24c | Describe and explain any amendments to information provided at registration or in the protocol. | NA |
| Support | 25 | Describe sources of financial or non-financial support for the review, and the role of the funders or sponsors in the review. | Funding Statement |
| Competing interests | 26 | Declare any competing interests of review authors. | Conflict of interest |
| Availability of data, code and other materials | 27 | Report which of the following are publicly available and where they can be found: template data collection forms; data extracted from included studies; data used for all analyses; analytic code; any other materials used in the review. | Data availability statement |

# Search strategy

Table S2 Search strategy

| **Pubmed**  ((("Non-alcoholic Fatty Liver Disease"[Mesh]) OR ((((((((Non-alcoholic Fatty Liver Disease) OR (Non alcoholic Fatty Liver Disease)) OR (Nonalcoholic Fatty Liver)) OR (Nonalcoholic Steatohepatitis)) OR (NAFLD)) OR (fatty liver)) OR (fatty liver disease)) OR (NASH)) OR (metabolic dysfunction-associated steatotic liver disease)) OR (MASLD)) OR (metabolic dysfunction-associated fatty liver disease)) OR (MAFLD)) OR (MASH))) AND ((((((("Diet, Carbohydrate-Restricted"[Mesh]) OR ((((Diet, Carbohydrate Restricted) OR (Carbohydrate-Restricted Diet)) OR (Low Carbohydrate Diet)) OR (LCD))) OR ("Dietary Carbohydrates"[Mesh])) OR ("Diet, Ketogenic"[Mesh])) OR (((Ketogenic Diet) OR (Ketogenic Diets)) OR (Keto*))) OR ("Diet, High-Protein Low-Carbohydrate"[Mesh])) OR ((((Diet, High Protein Low Carbohydrate) OR (High-Protein Low-Carbohydrate Diet)) OR (Atkins Diet)) OR (South Beach Diet)))) AND (("Randomized Controlled Trial" [Publication Type]) OR (((((controlled clinical trial) OR (RCT)) OR (random*)) OR (placebo)) OR (blind))) |
| --- |
| **Cochrane library**  #1 MeSH descriptor: [Non-alcoholic Fatty Liver Disease] explode all trees  #2 (Non alcoholic Fatty Liver Disease):ti,ab,kw OR (Nonalcoholic Fatty Liver):ti,ab,kw OR (Nonalcoholic Steatohepatitis):ti,ab,kw OR (NAFLD):ti,ab,kw OR (fatty liver):ti,ab,kw OR (fatty liver disease):ti,ab,kw OR (NASH):ti,ab,kw OR (metabolic dysfunction-associated steatotic liver disease):ti,ab,kw OR (MASLD):ti,ab,kw OR (metabolic dysfunction-associated fatty liver disease):ti,ab,kw OR (MAFLD):ti,ab,kw OR (MASH):ti,ab,kw  #3 #1 OR #2  #4 MeSH descriptor: [Diet, Carbohydrate-Restricted] explode all trees  #5 (Diet, Carbohydrate Restricted):ti,ab,kw OR (Carbohydrate-Restricted Diet):ti,ab,kw OR (Low Carbohydrate Diet):ti,ab,kw OR (LCD):ti,ab,kw  #6 MeSH descriptor: [Dietary Carbohydrates] explode all trees  #7 MeSH descriptor: [Diet, Ketogenic] explode all trees  #8 (Diet, Ketogenic):ti,ab,kw OR (Ketogenic Diet):ti,ab,kw OR (Ketogenic Diets):ti,ab,kw OR (Keto*):ti,ab,kw  #9 MeSH descriptor: [Diet, High-Protein Low-Carbohydrate] explode all tree  #10 (Diet, High Protein Low Carbohydrate):ti,ab,kw OR (High-Protein Low-Carbohydrate Diet):ti,ab,kw OR (Atkins Diet):ti,ab,kw OR (South Beach Diet):ti,ab,k  #11 #4 OR #5 OR #6 OR #7 OR #8 OR #9 OR #10  #12 MeSH descriptor: [Randomized Controlled Trial] explode all tree  #13 (controlled clinical trial):ti,ab,kw OR (RCT):ti,ab,kw OR (random*):ti,ab,kw OR (placebo):ti,ab,kw OR (blind):ti,ab,kw  #14 #12 OR #13  #15 #3 AND #11 AND #14 |
| **Web of Science**  #1 TS=(Non-alcoholic Fatty Liver Disease OR Non alcoholic Fatty Liver Disease OR Nonalcoholic Fatty Liver OR Nonalcoholic Steatohepatitis OR NAFLD OR fatty liver OR fatty liver disease OR NASH OR metabolic dysfunction-associated steatotic liver disease OR MASLD OR metabolic dysfunction-associated fatty liver disease OR MAFLD OR MASH)  #2 TS=(Diet, Carbohydrate-Restricted OR Diet, Carbohydrate Restricted OR Carbohydrate-Restricted Diet OR Low Carbohydrate Diet OR LCD OR Dietary Carbohydrates OR Diet, Ketogenic OR Ketogenic Diet OR Ketogenic Diets OR Keto* OR Diet, High-Protein Low-Carbohydrate OR Diet, High Protein Low Carbohydrate OR High-Protein Low-Carbohydrate Diet OR Atkins Diet OR South Beach Diet)  #3 TS=(Randomized Controlled Trial OR controlled clinical trial OR RCT OR random* OR placebo OR blind)  #1 AND #2 AND #3 |
| **Scopus**  ( ( TITLE-ABS-KEY ( non-alcoholic AND fatty AND liver AND disease )OR TITLE-ABS-KEY ( non AND alcoholic AND fatty AND liver AND disease )OR TITLE-ABS-KEY ( nonalcoholic AND fatty AND liver )OR TITLE-ABS-KEY ( nonalcoholic AND steatohepatitis )OR TITLE-ABS-KEY ( nafld )OR TITLE-ABS-KEY ( fatty AND liver )OR TITLE-ABS-KEY ( fatty AND liver AND disease )OR TITLE-ABS-KEY ( nash )OR TITLE-ABS-KEY (metabolic dysfunction-associated steatotic liver disease )OR TITLE-ABS-KEY ( MASLD )OR TITLE-ABS-KEY (metabolic dysfunction-associated fatty liver disease )OR TITLE-ABS-KEY ( MAFLD )OR TITLE-ABS-KEY ( MASH ) ) ) AND ( ( TITLE-ABS-KEY ( diet, AND carbohydrate-restricted )OR TITLE-ABS-KEY ( diet, AND carbohydrate AND restricted )OR TITLE-ABS-KEY ( carbohydrate-restricted AND diet )OR TITLE-ABS-KEY ( low AND carbohydrate AND diet )OR TITLE-ABS-KEY ( lcd )OR TITLE-ABS-KEY ( dietary AND carbohydrates )OR TITLE-ABS-KEY ( diet, AND ketogenic )OR TITLE-ABS-KEY ( ketogenic AND diet )OR TITLE-ABS-KEY ( ketogenic AND diets )OR TITLE-ABS-KEY ( keto* )OR TITLE-ABS-KEY ( diet, AND high-protein AND low-carbohydrate )OR TITLE-ABS-KEY ( diet, AND high AND protein AND low AND carbohydrate )OR TITLE-ABS-KEY ( high-protein AND low-carbohydrate AND diet )OR TITLE-ABS-KEY ( atkins AND diet )OR TITLE-ABS-KEY ( south AND beach AND diet ) ) ) AND ( ( TITLE-ABS-KEY ( randomized AND controlled AND trial )OR TITLE-ABS-KEY ( controlled AND clinical AND trial )OR TITLE-ABS-KEY ( rct )OR TITLE-ABS-KEY ( random* )OR TITLE-ABS-KEY ( placebo )OR TITLE-ABS-KEY ( blind ) ) ) |

# Subgroup analysis stratified by dietary carbohydrate intake

**
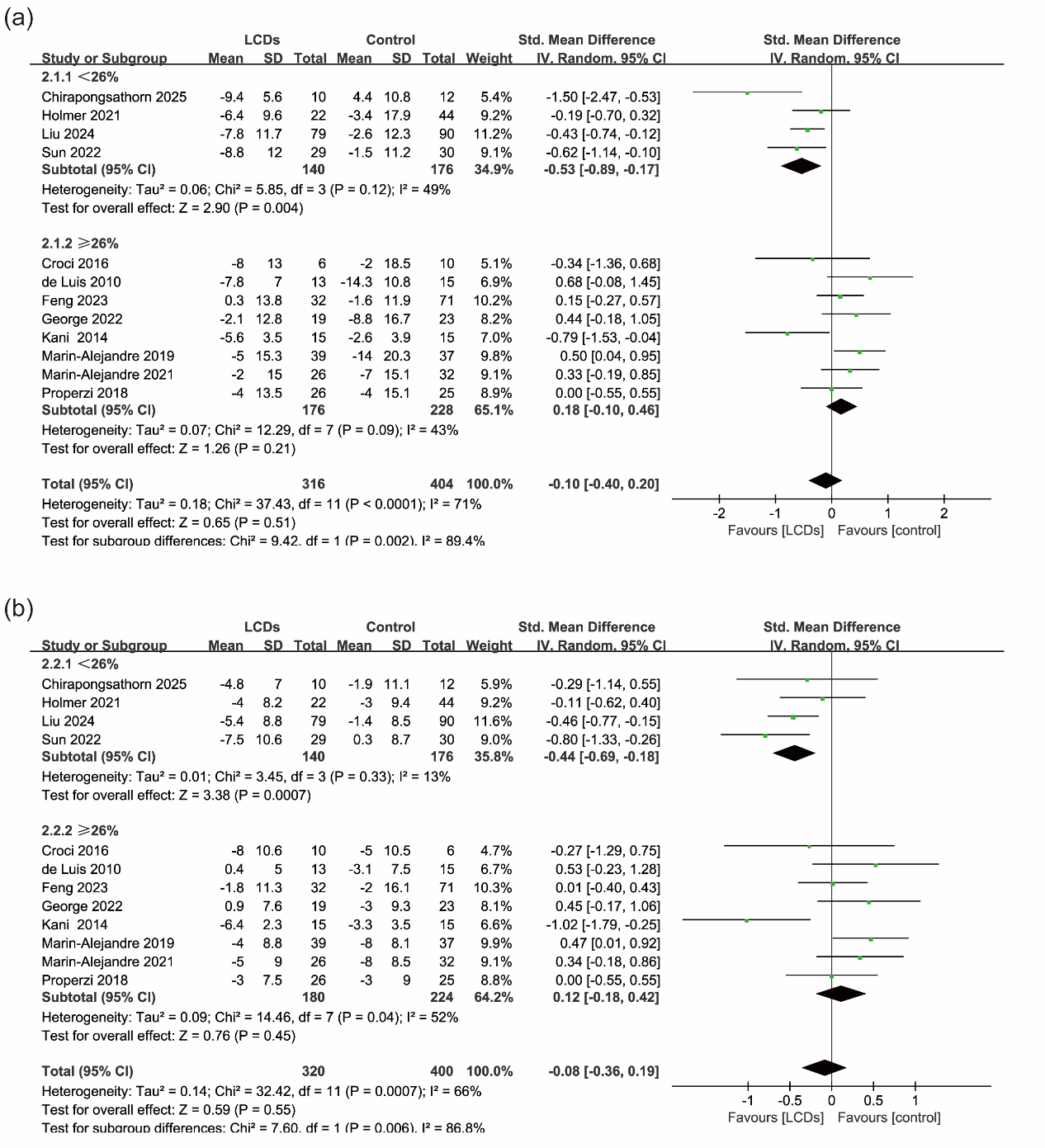
**

Figure S1 Subgroup analysis for (a) SBP and (b) DBP. SBP, systolic blood pressure; DBP, diastolic blood pressure.

**
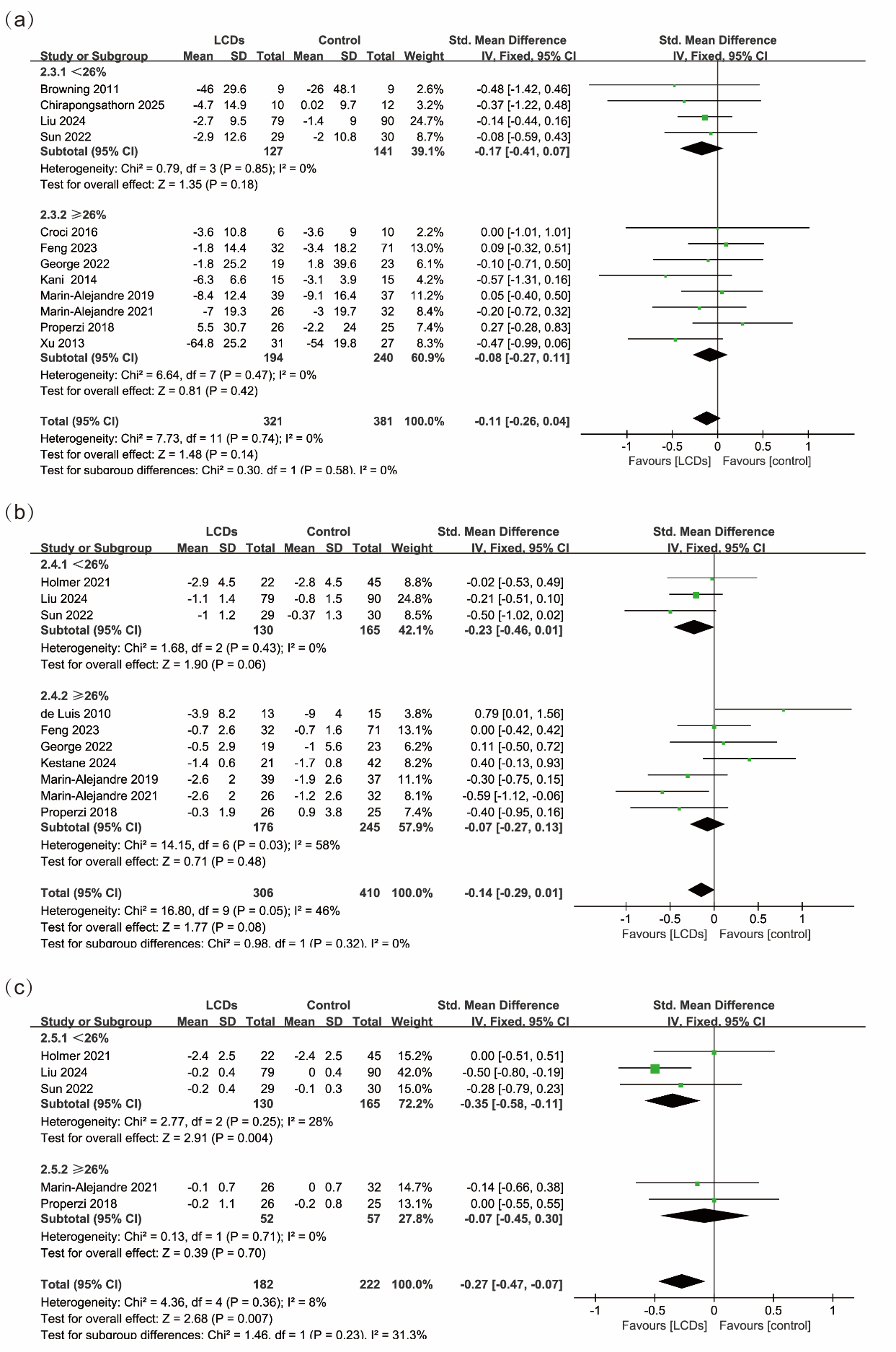
**

Figure S2 Subgroup analysis for (a) FBG, (b) HOMA-IR, and (c) HbA1c. FBG, fasting blood glucose; HOMA-IR, homeostatic model assessment insulin resistance index; HbA1c, glycated hemoglobin.

**
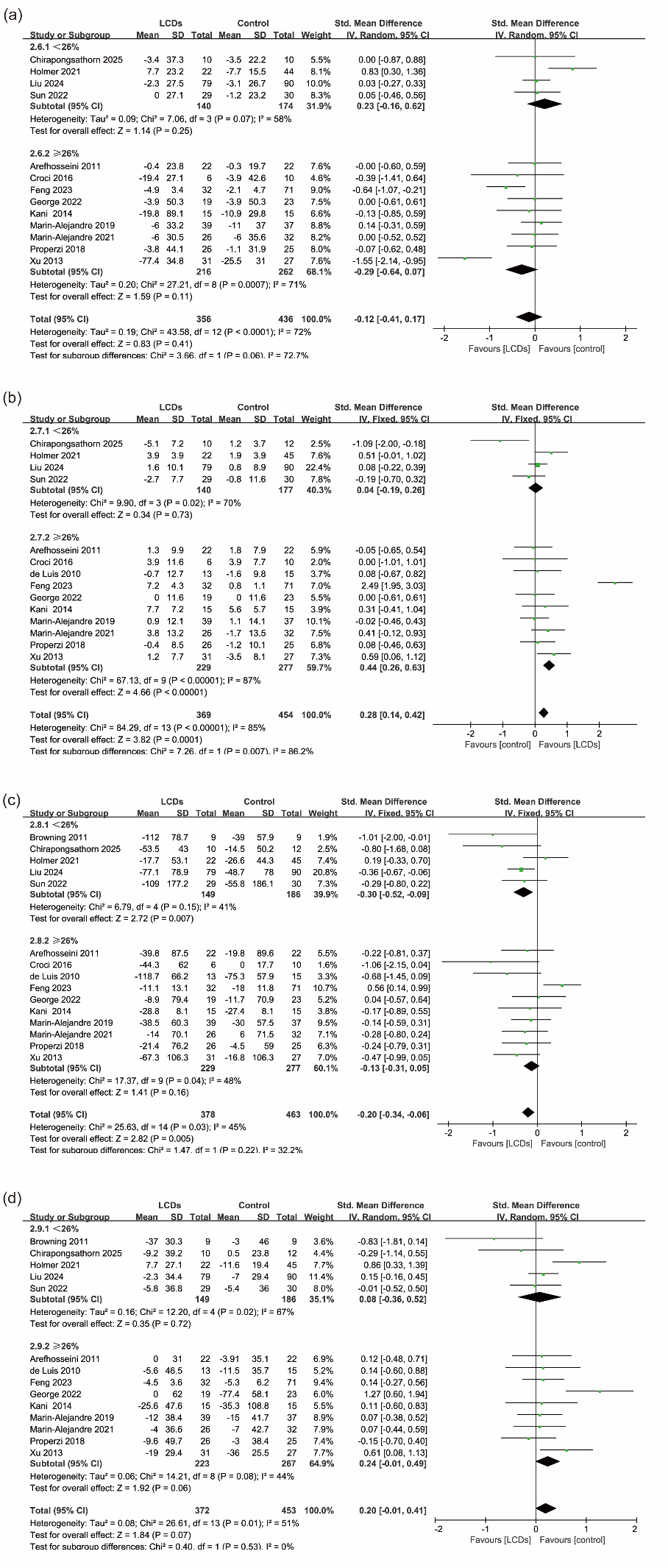
**

Figure S3 Subgroup analysis for (a) LDL-C, (b) HDL-C, (c) TG, and (d) TC. LDL-C, low-density lipoprotein cholesterol; HDL-C, high-density lipoprotein cholesterol; TG, triglyceride; TC, total cholesterol.

**
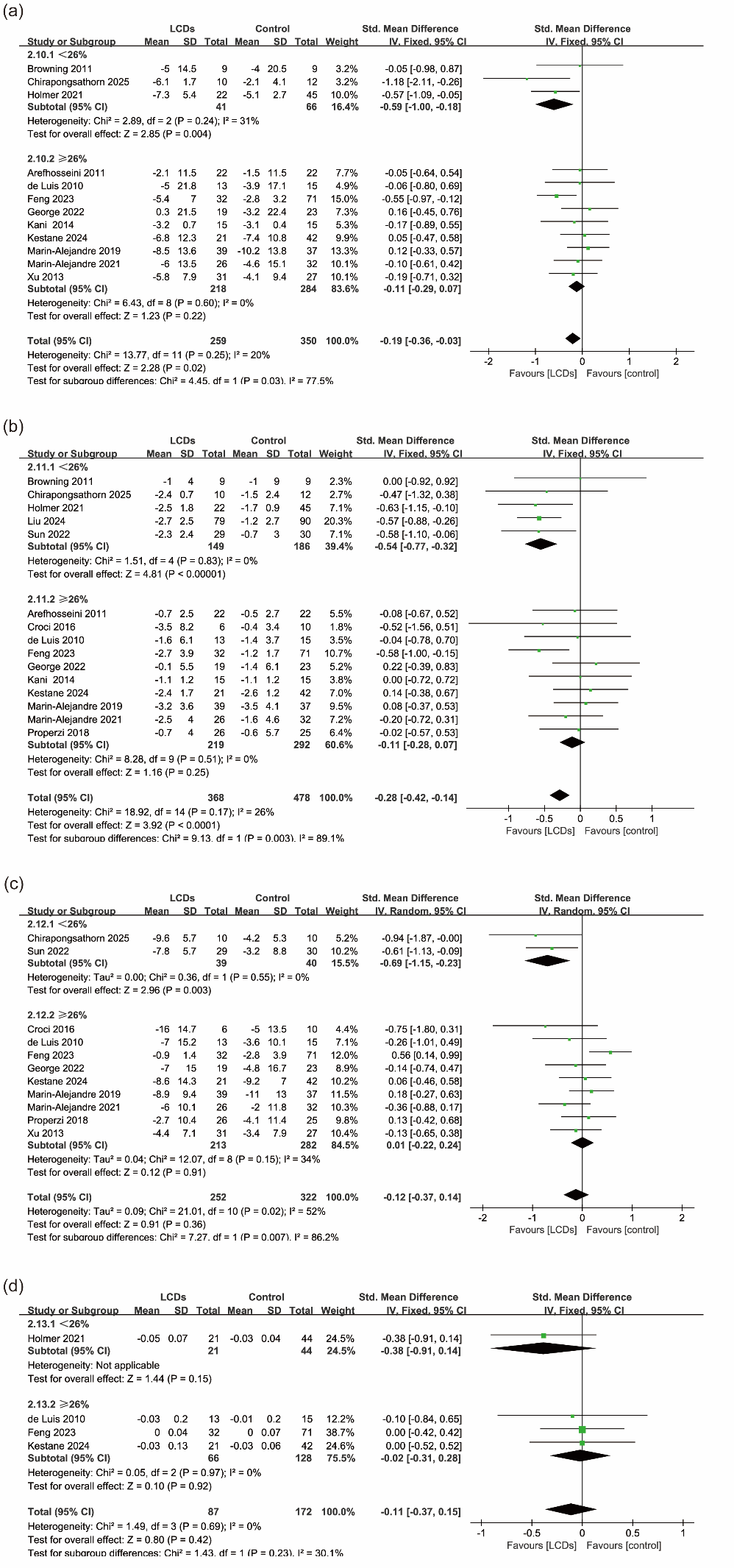
**

Figure S4 Subgroup analysis for (a) body weight, (b) BMI, (c) WC, and (d) WHR. BMI, body mass index; WC, waist circumference; WHR, waist-hip ratio.

# Subgroup analysis stratified by intervention duration

**
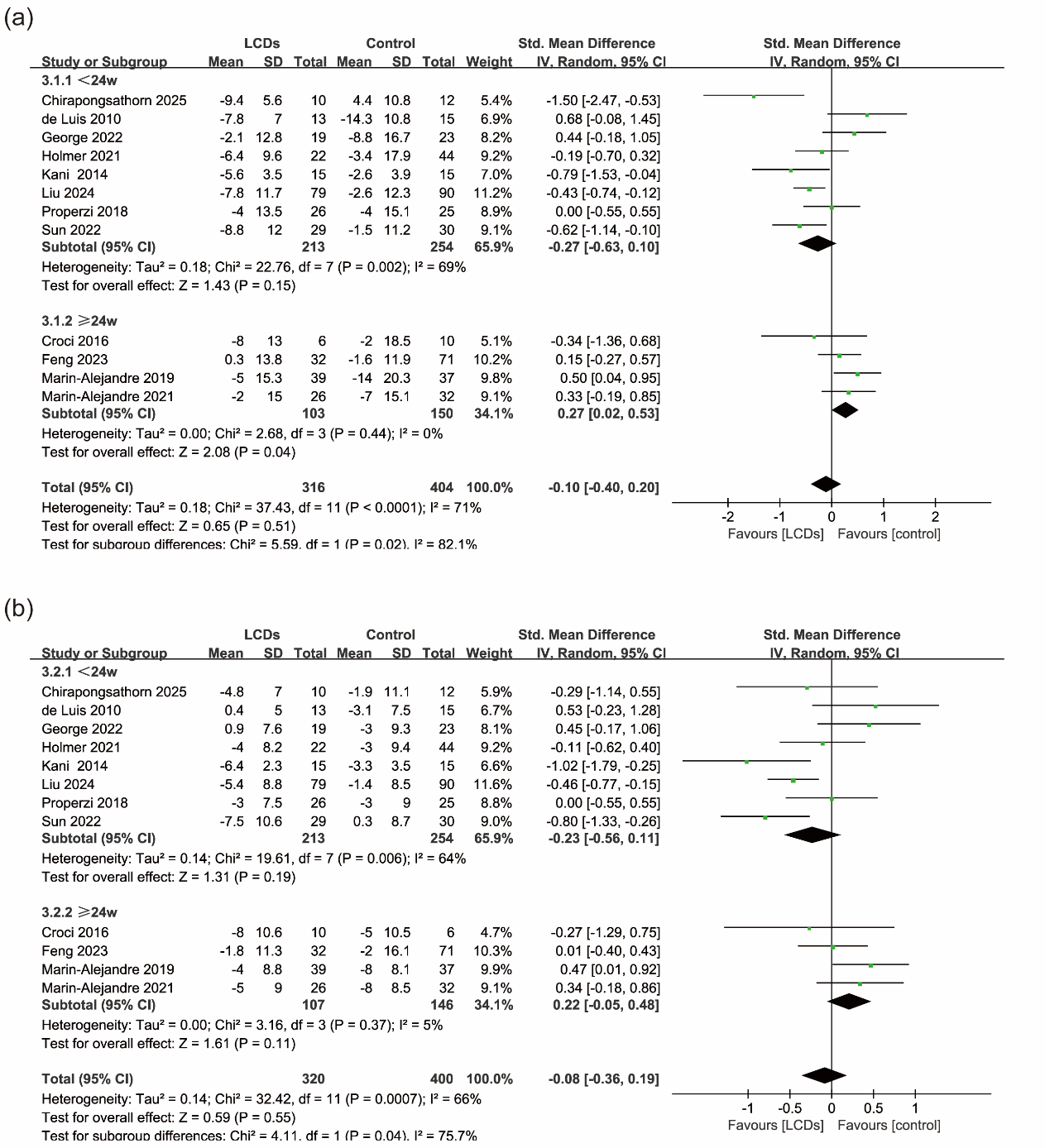
**

Figure S5 Subgroup analysis for (a) SBP and (b) DBP. SBP, systolic blood pressure; DBP, diastolic blood pressure.

**
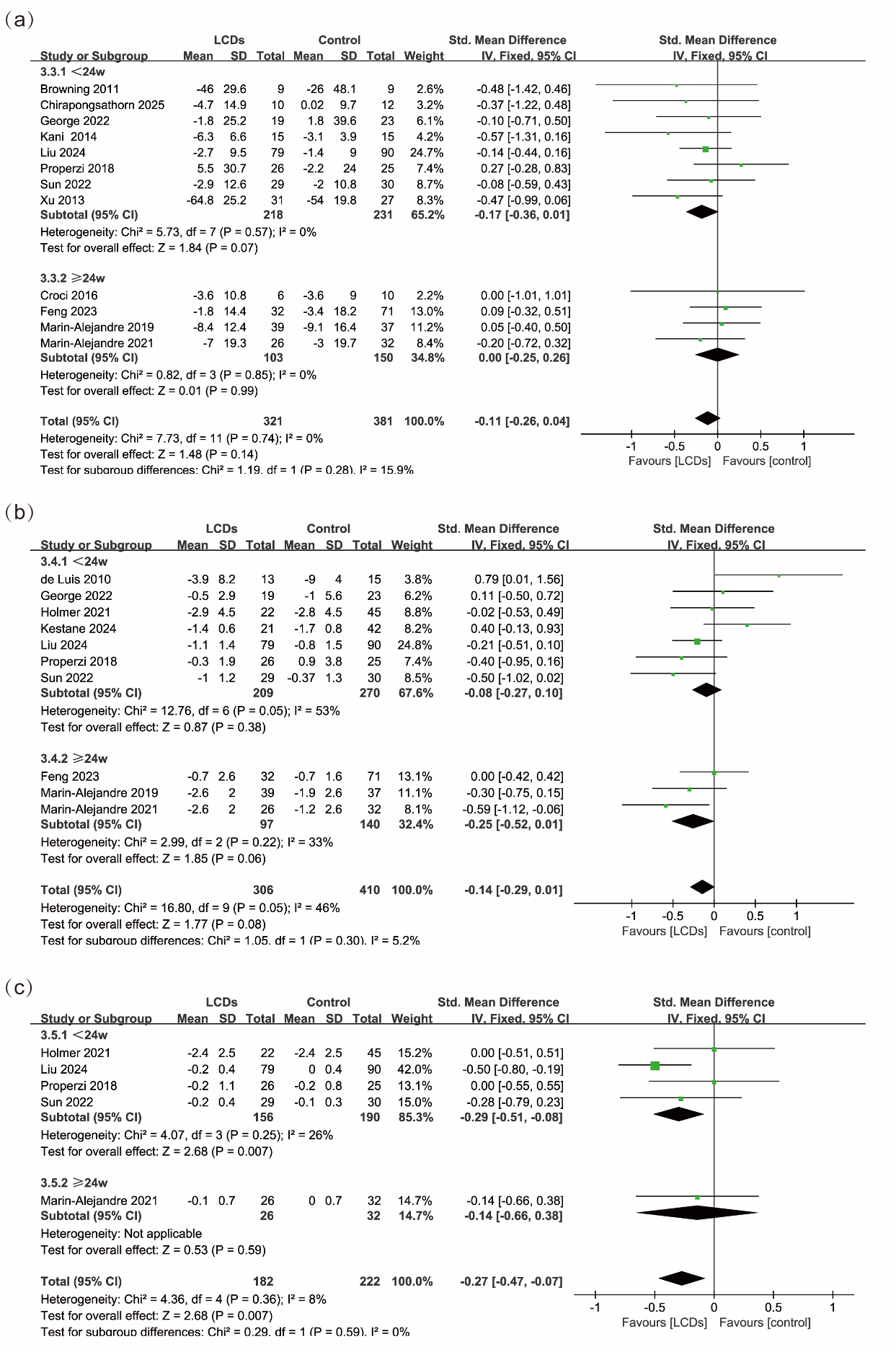
**

Figure S6 Subgroup analysis for (a) FBG, (b) HOMA-IR, and (c) HbA1c. FBG, fasting blood glucose; HOMA-IR, homeostatic model assessment insulin resistance index; HbA1c, glycated hemoglobin.

**
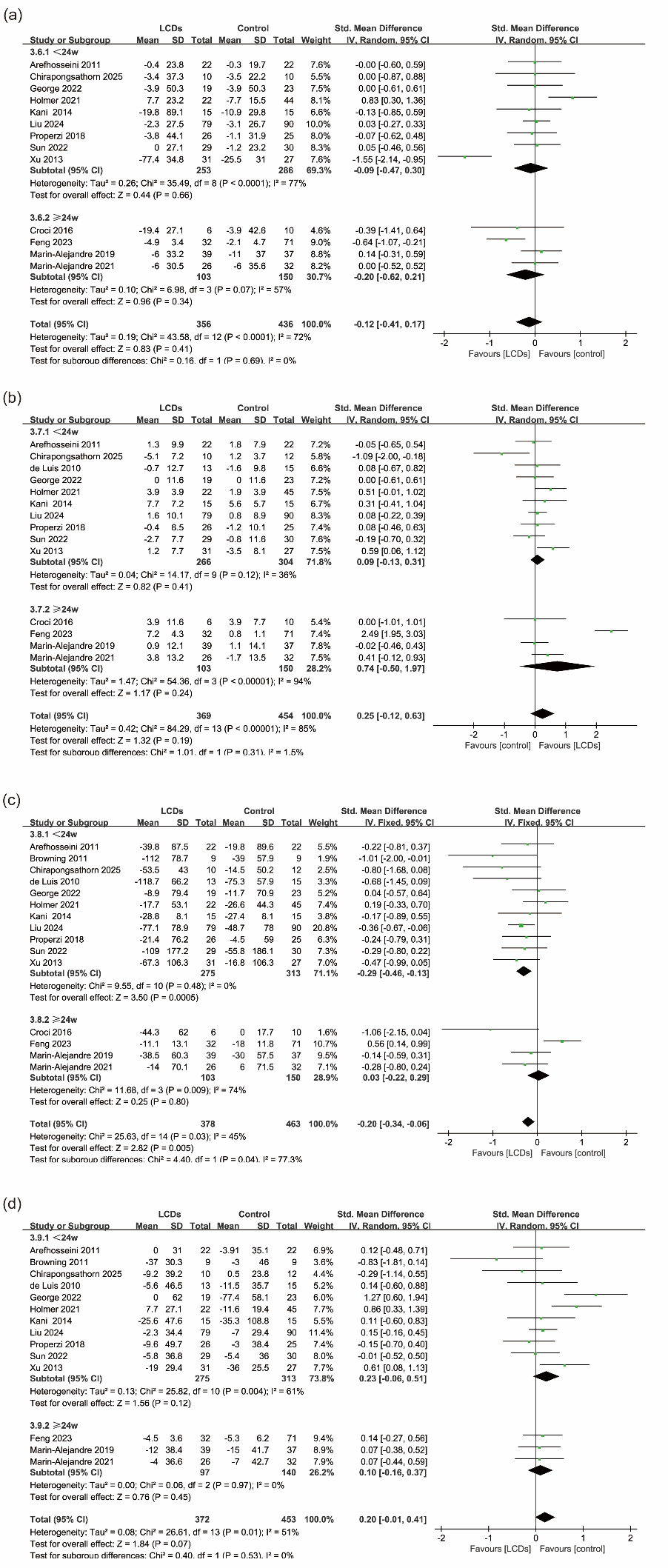
**

Figure S7 Subgroup analysis for (a) LDL-C, (b) HDL-C, (c) TG, and (d) TC. LDL-C, low-density lipoprotein cholesterol; HDL-C, high-density lipoprotein cholesterol; TG, triglyceride; TC, total cholesterol.

**
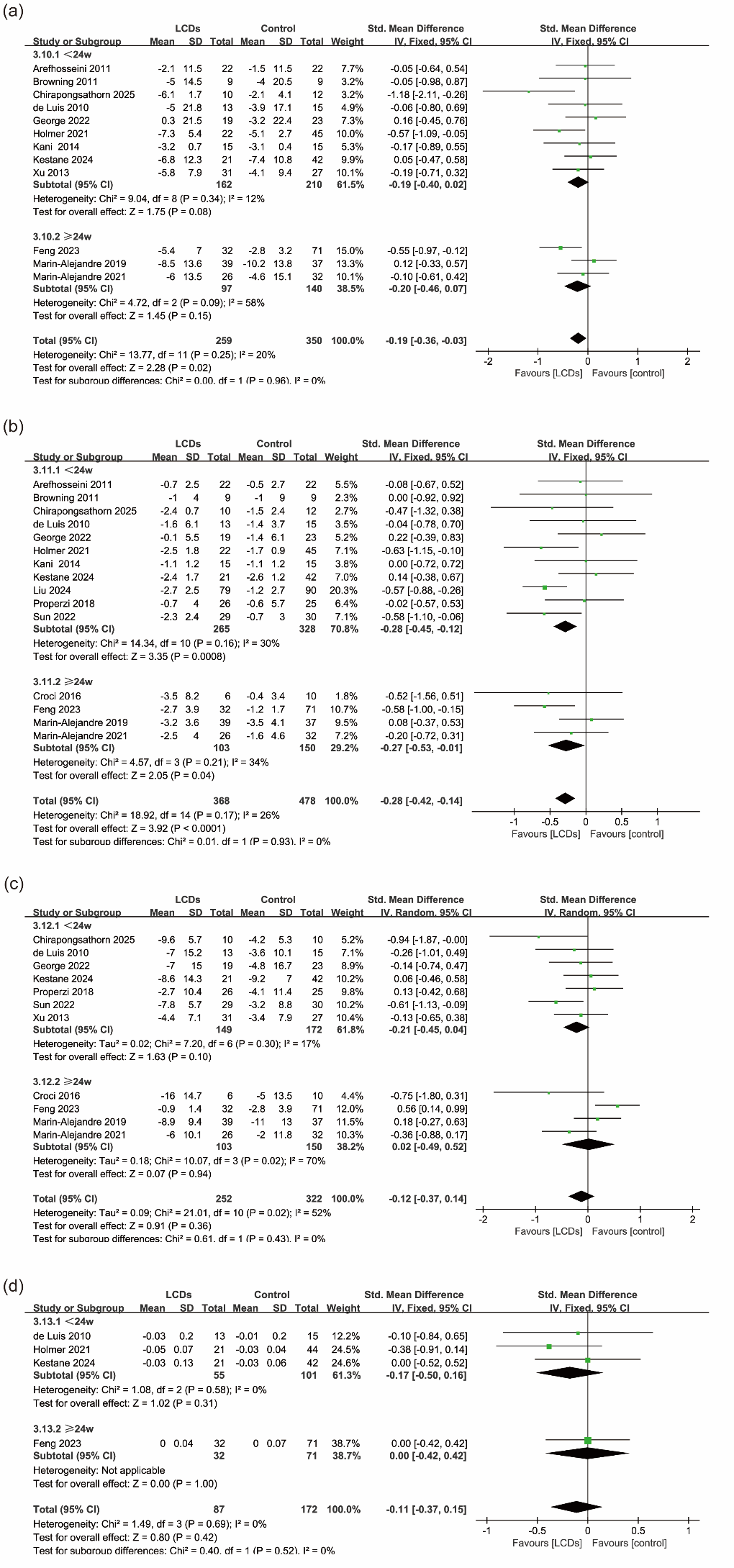
**

Figure S8 Subgroup analysis for (a) body weight, (b) BMI, (c) WC, and (d) WHR. BMI, body mass index; WC, waist circumference; WHR, waist-hip ratio.

# Funnel plots

**
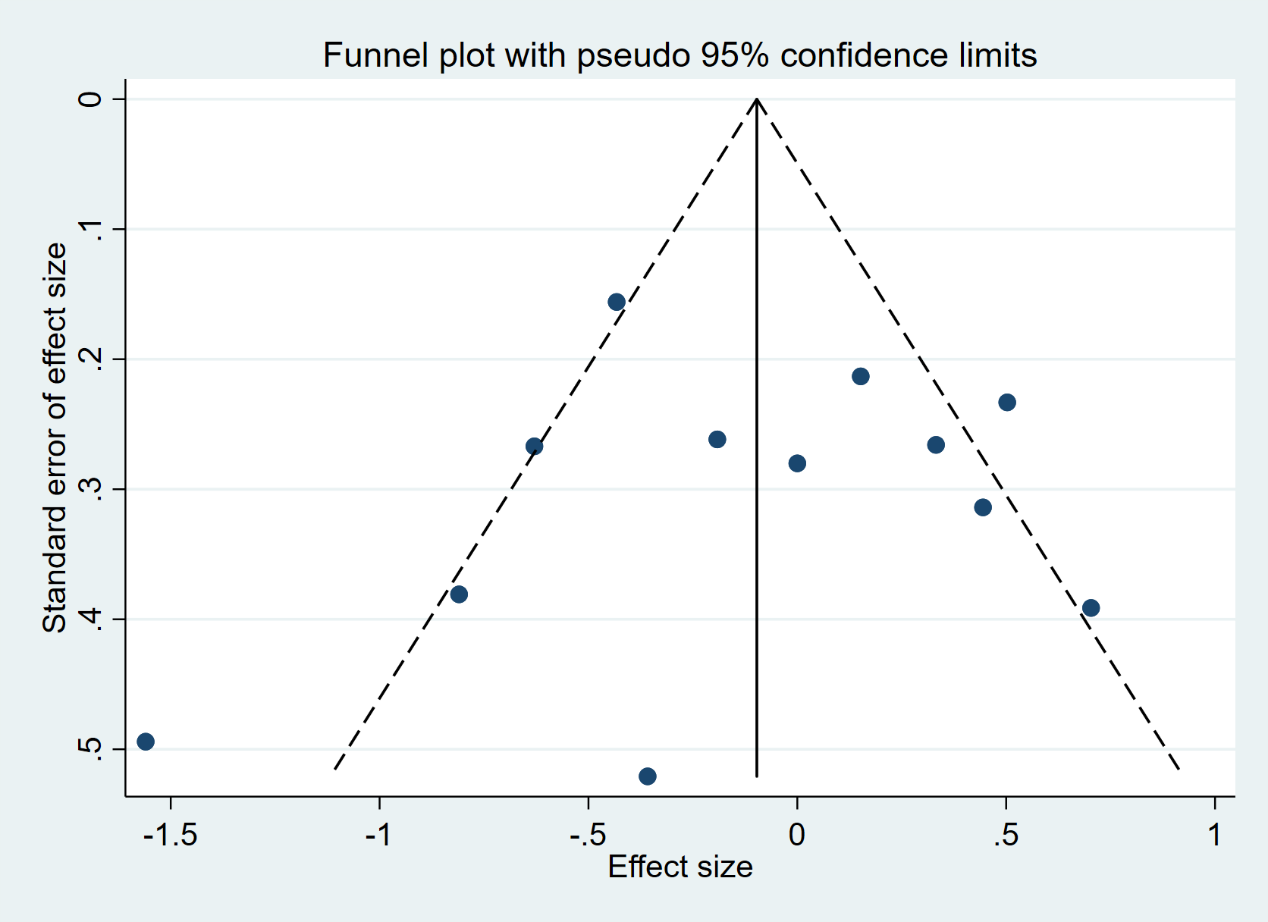
**

Figure S9a Funnel plot for systolic blood pressure.

**
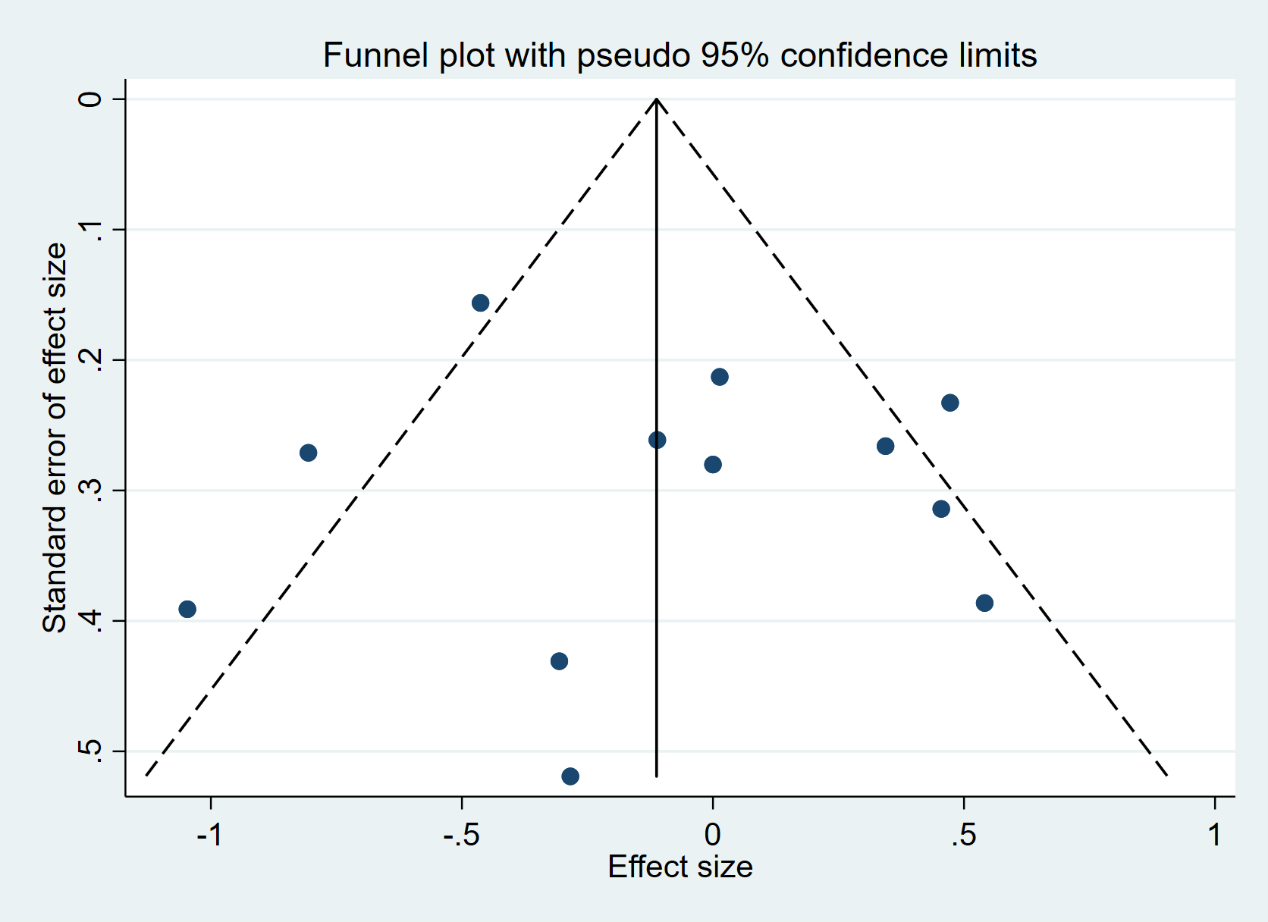
**

Figure S9b Funnel plot for diastolic blood pressure.

**
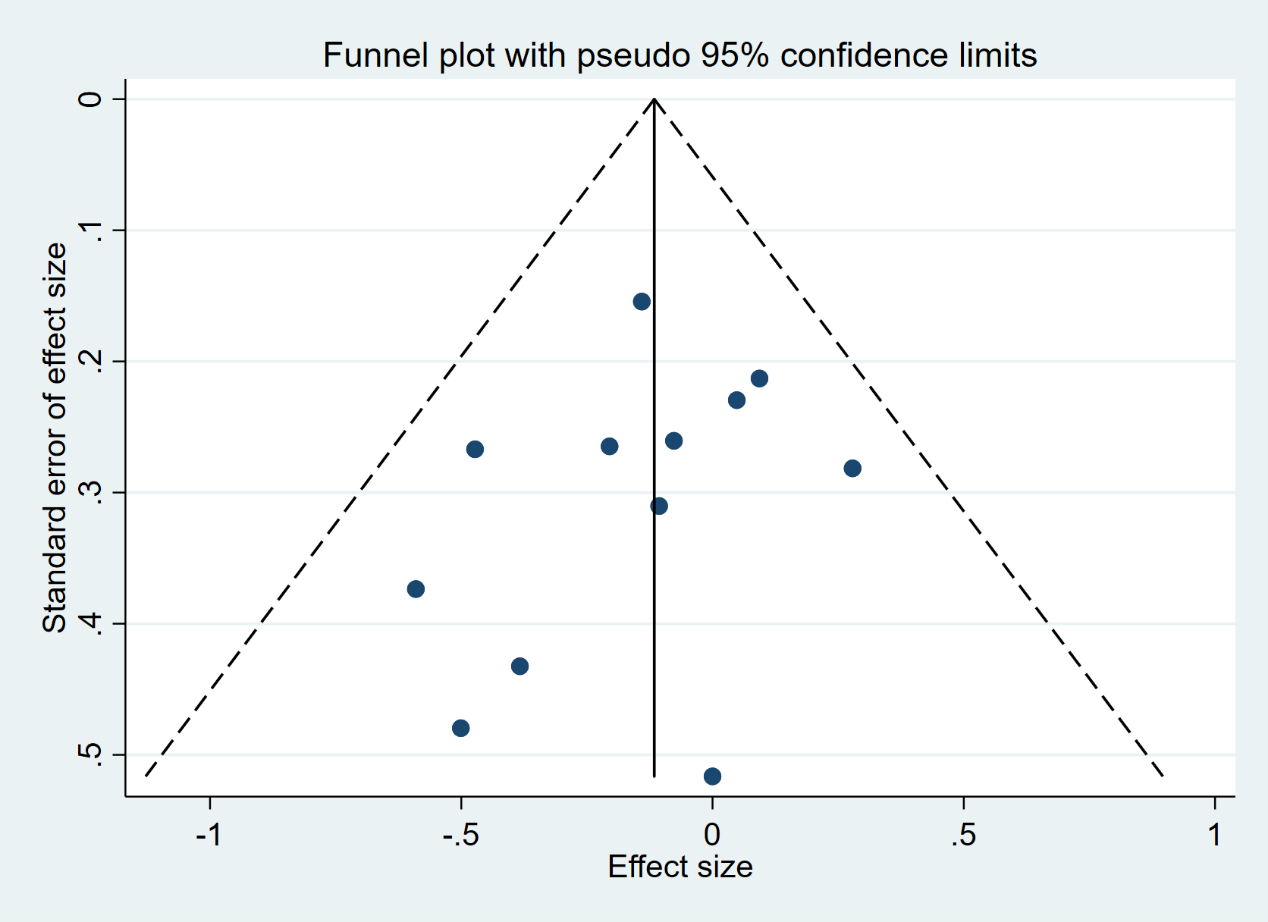
**

Figure S9c Funnel plot for fasting blood glucose.


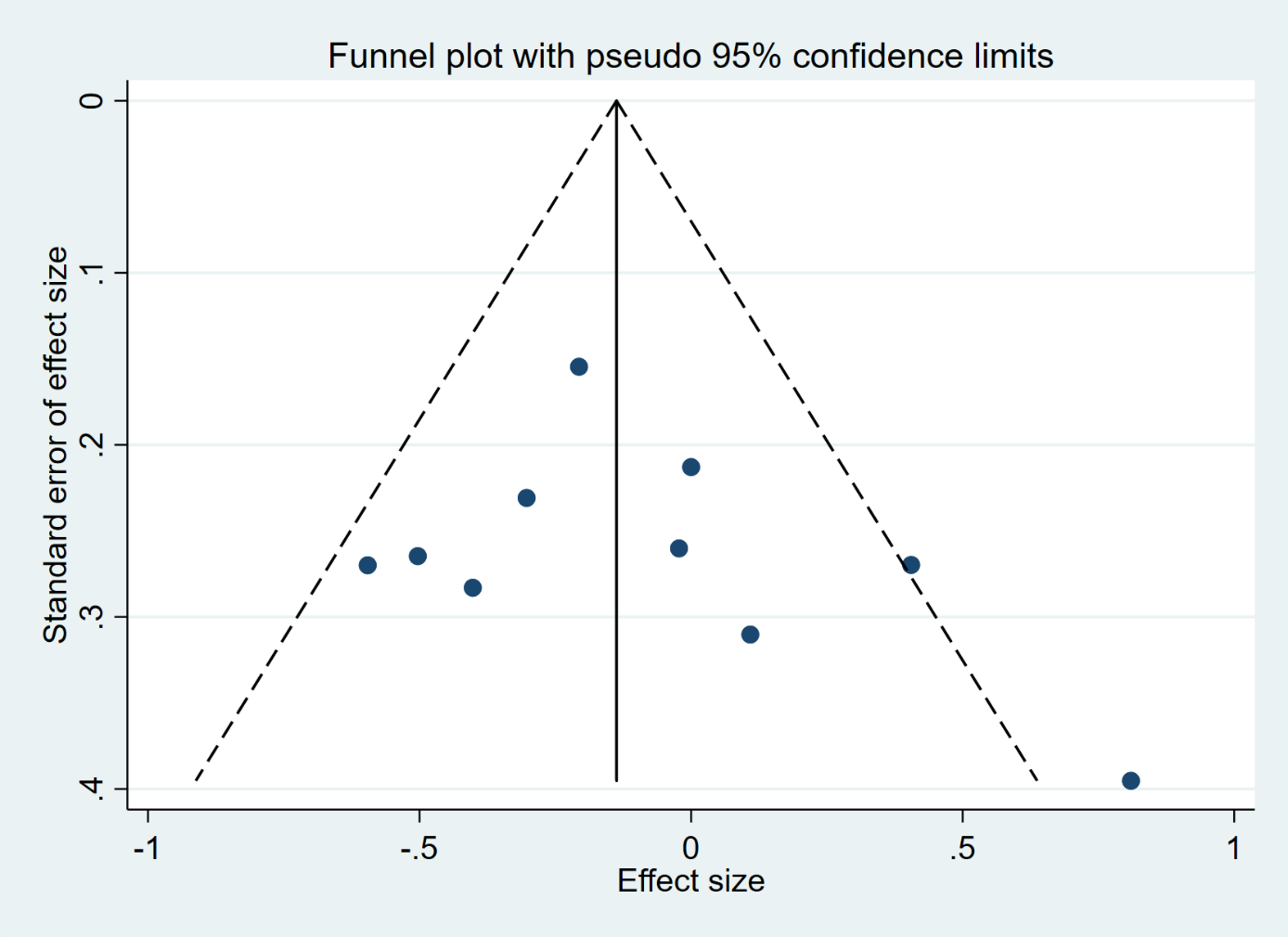


Figure S9d Funnel plot for homeostatic model assessment insulin resistance index.

**
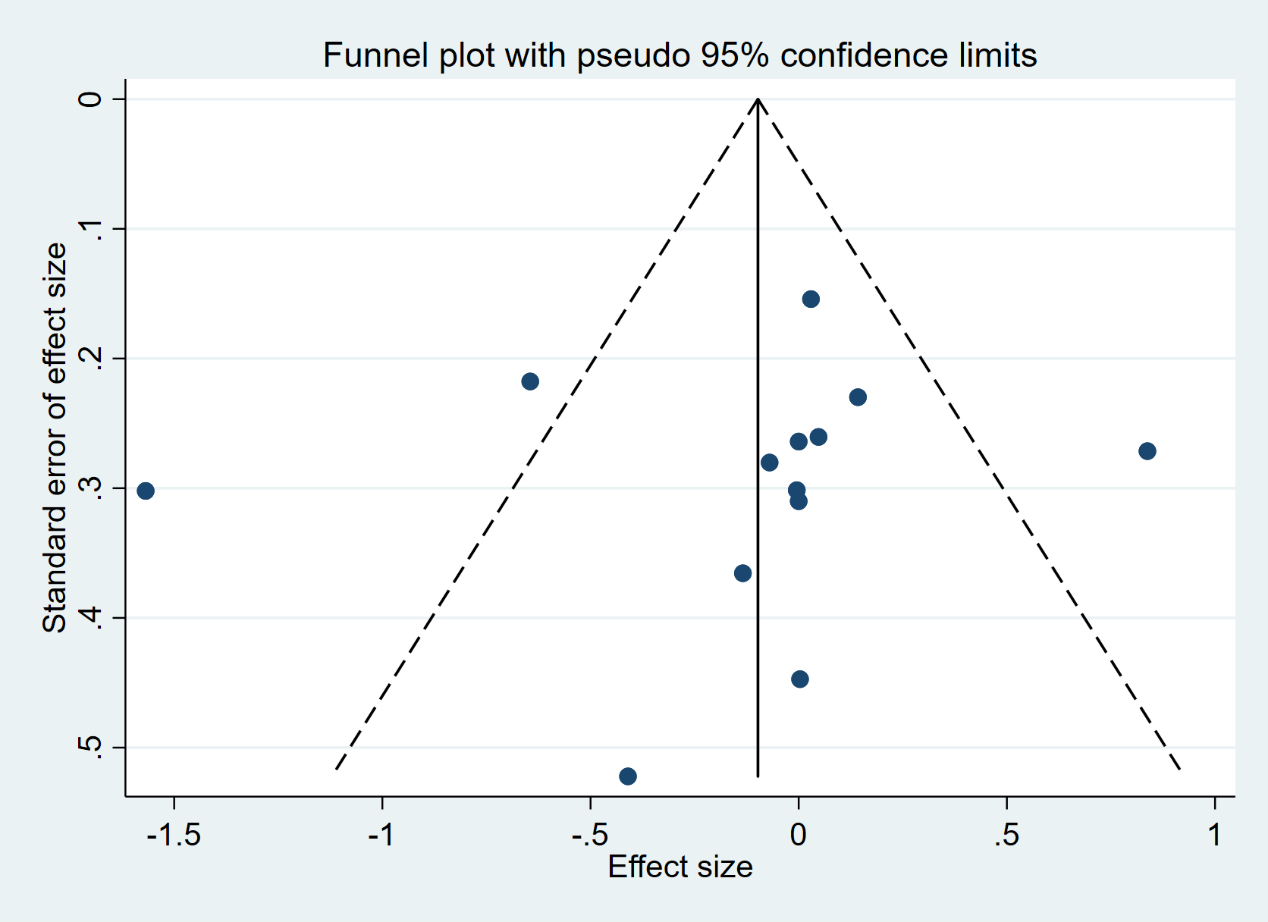
**

Figure S9e Funnel plot for low-density lipoprotein cholesterol.

**
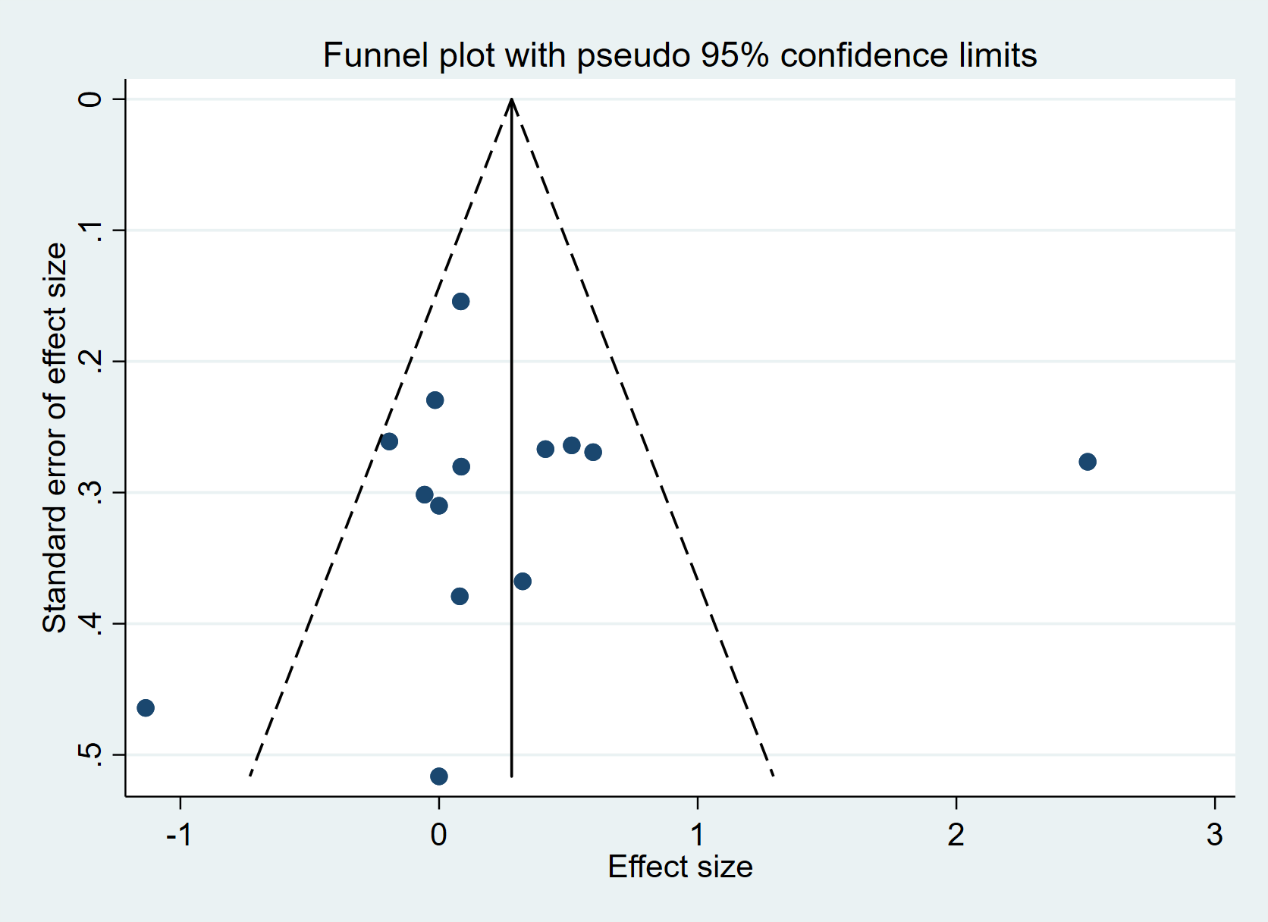
**

Figure S9f Funnel plot for high-density lipoprotein cholesterol.

**
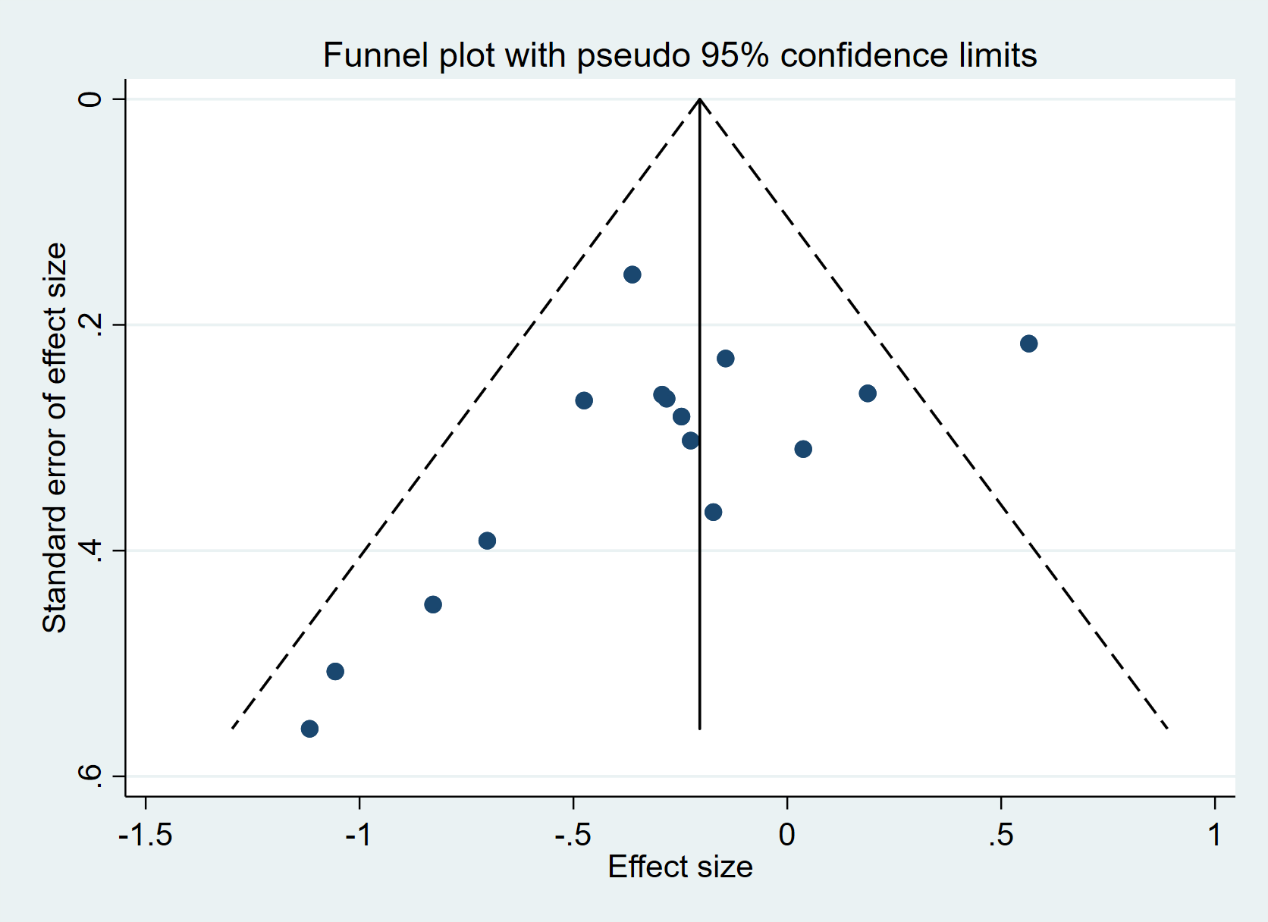
**

Figure S9g Funnel plot for triglyceride.

**
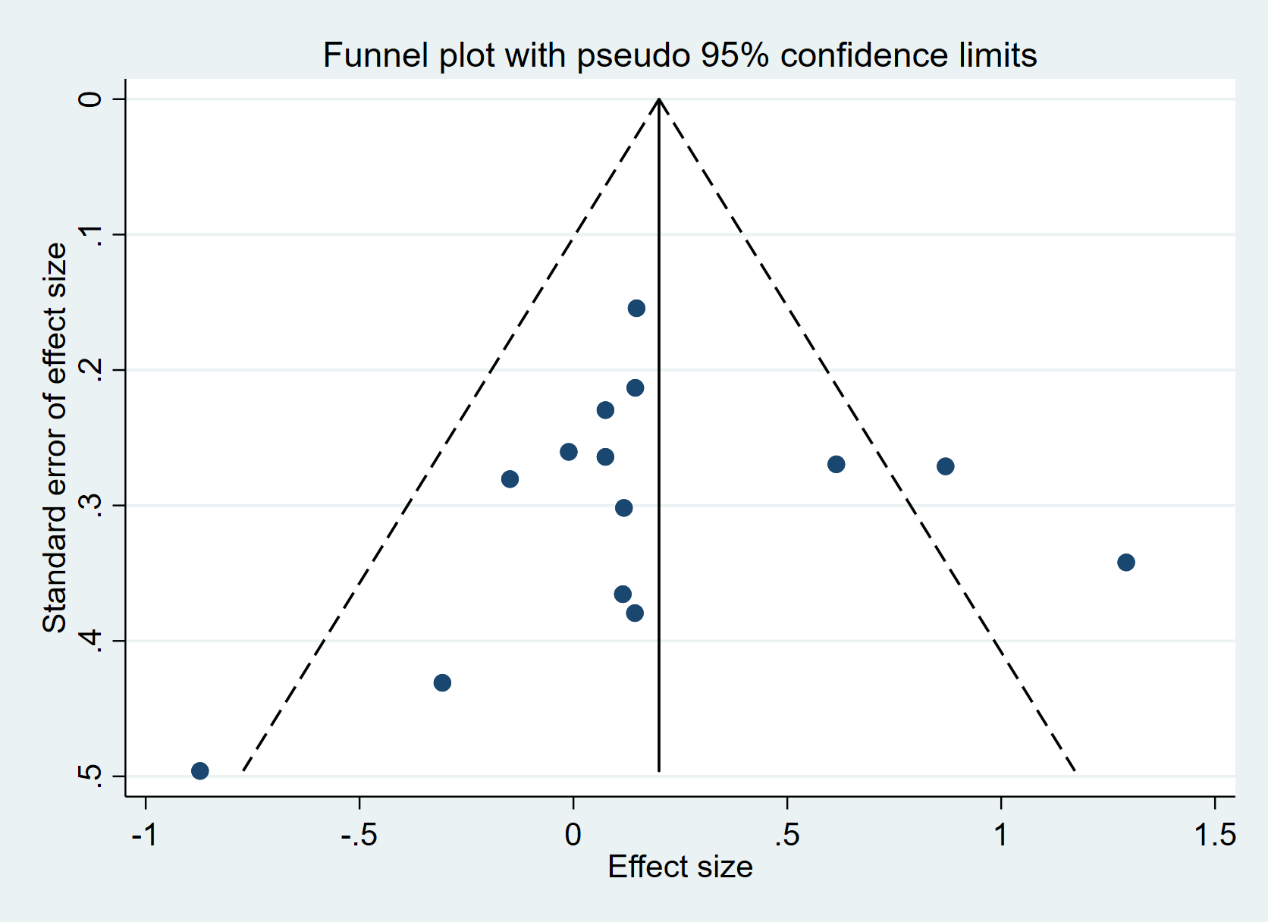
**

Figure S9h Funnel plot for total cholesterol.

**
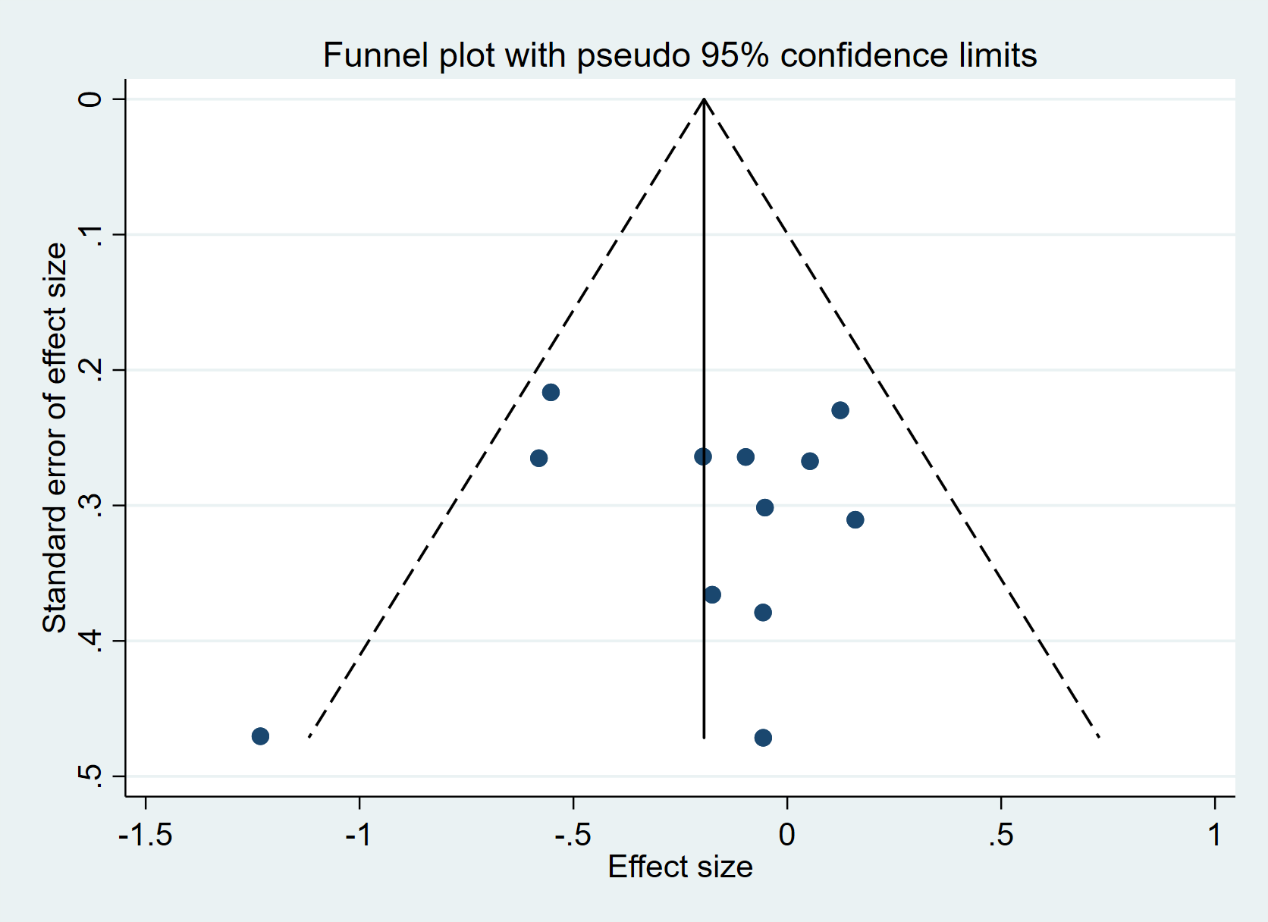
**

Figure S9i Funnel plot for body weight.

**
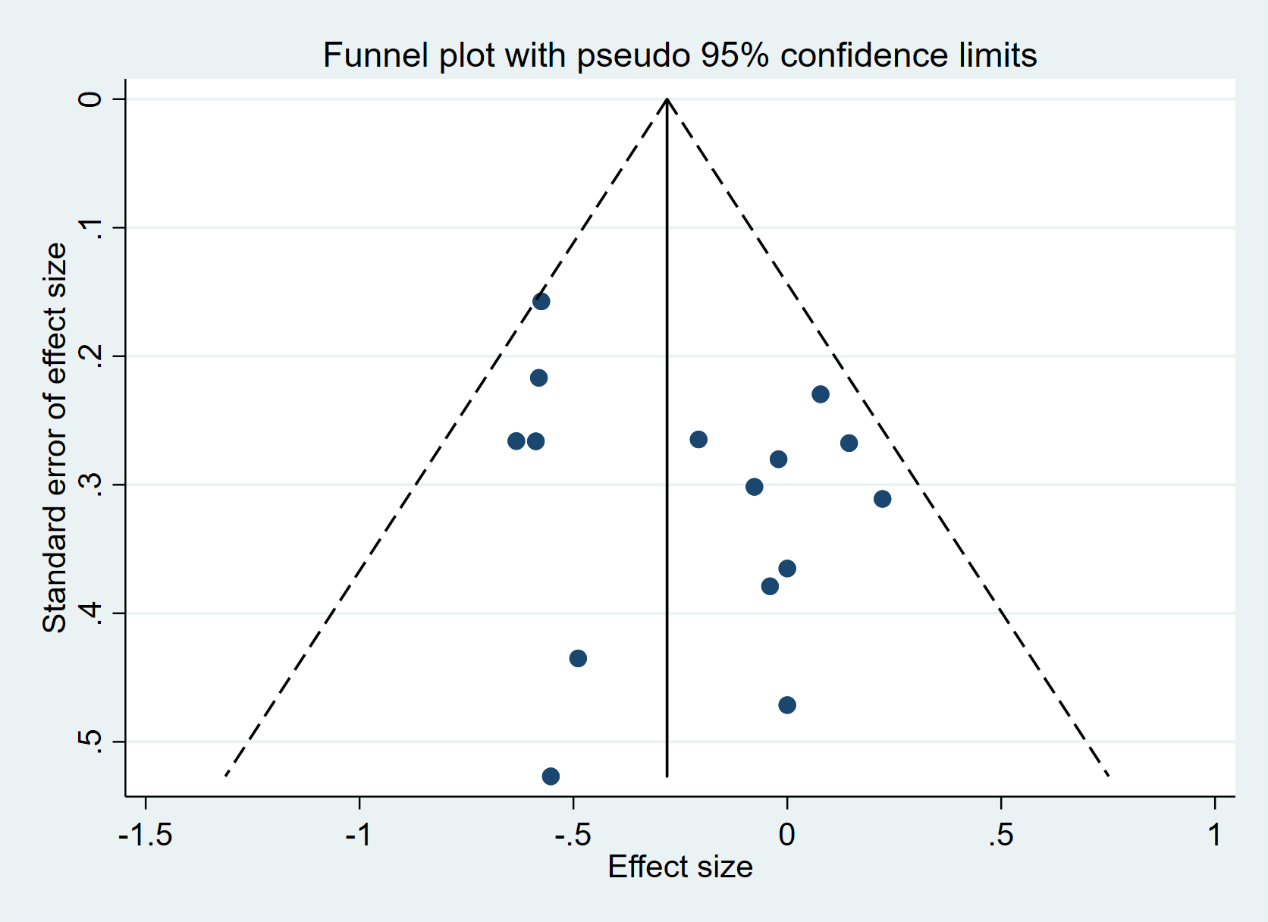
**

Figure S9j Funnel plot for body mass index.


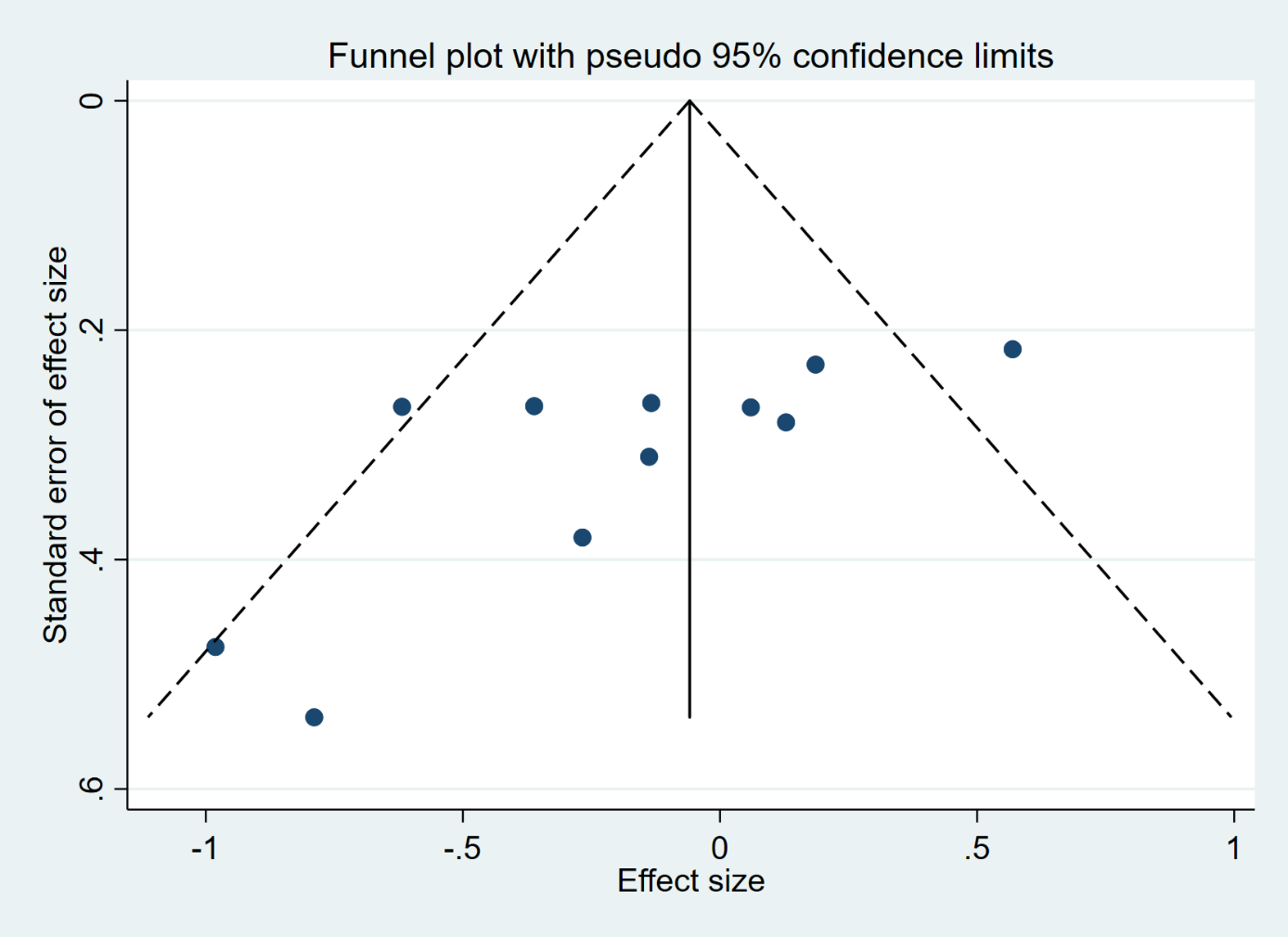


Figure S9k Funnel plot for waist circumference.

# Dietary Fat Composition

Table S3 Dietary fat composition in included studies.

| Study | Total Fat (%E: C/I) | SFA (%E: C/I) | MUFA (%E: C/I) | PUFA (%E: C/I) |
| --- | --- | --- | --- | --- |
| de Luis et al. | NA | NA | NA | NA |
| Arefhosseini et al. | NA | NA | NA | NA |
| Browning et al. | 34/59 | 14.3/21.8 | 12.6/22.4 | 6.1/8.9 |
| Xu et al. | NA | NA | NA | NA |
| Kani et al. | 30.3/36.7 | 9.0/9.9 | 7.5/7.4 | NA |
| Croci et al. | NA | NA | NA | NA |
| Properzi et al. | 30.6/44.7 | 9.3/9.5 | 12.4/23.9 | NA |
| Marin-Alejandre et al. | 34.5/36.5 | 9.4/8.8 | 17.5/16.1 | 5.2/9.0 |
| Holmer et al. | NA | NA | NA | NA |
| Marin-Alejandre et al. 2021 | 37.0/37.3 | 10.3/8.6 | 18.8/17.6 | 6.1/8.8 |
| George et al. | 32.7/40.1 | 10.3/10.8 | 13.4/19.0 | 5.8/7.7 |
| Sun et al. | 41.3/46.2 | NA | NA | NA |
| Feng et al. | 19.5/26.4 | NA | NA | NA |
| Liu et al. | 39.9/45.2 | NA | NA | NA |
| Uluçay Kestane and Baş | 31.8/47.1 | 6.1/8.9 | 17.1/25.9 | 6.7/9.5 |
| Chirapongsathorn et al. | NA | NA | NA | NA |

Abbreviations: %E, percentage of total energy intake; C/I, control/intervention; SFA, saturated fatty acids; MUFA, monounsaturated fatty acids; PUFA, polyunsaturated fatty acids.
